# Immuno-diagnosis of *Mycobacterium tuberculosis* in sputum, and reduction of timelines for its positive cultures to within 3 h by pathogen-specific thymidylate kinase expression assays

**DOI:** 10.1186/s13104-017-2649-y

**Published:** 2017-08-08

**Authors:** Misaki Wayengera, Ivan Mwebaza, Johnson Welishe, Alice Bayiyana, David P. Kateete, Eddie Wampande, Samuel Kirimunda, Edgar Kigozi, Fred Katabazi, Carol Musubika, Samuel Kyobe, Peace Babirye, Benon Asiimwe, Moses L. Joloba

**Affiliations:** 1 Unit of Genetics & Genomics, Dept. of Pathology, School of Biomedical Science, Makerere University College of Health Sciences, P o Box 7072, Kampala, Uganda; 2 Dept of Immunology/Molecular Biology (Mycobacteriology Laboratory), School of Biomedical Sciences, Makerere University College of Health Sciences, P o Box 7072, Kampala, Uganda; 3 Dept. of Medical Microbiology, School of Biomedical Sciences, Makerere University College of Health Sciences, P o Box 7072, Kampala, Uganda

**Keywords:** Tuberculosis, Immunodiagnosis, *Mycobacteriumtuberculosis*, Thymidylate Kinase, Sputum, Cultures

## Abstract

**Background:**

Laboratory diagnosis of Tuberculosis (TB) is traditionally based on microscopy and or culture. Microscopy is however, only sensitive to a specified degree of bacillary load not present in HIV co-infected persons. Traditional cultures of *Mycobacterium tuberculosis* (*M*. *tb*), *on the other hand,* take weeks to read—thereby delaying the critical decision whether or not, to treat. Although nucleic acids amplification tests (NAATS) applied directly on sputum or cultures can increase the sensitivity for TB diagnosis among those with HIV co-infection as well as reduce time-lines for positive culture detection, they do not replace the need for smear microscopy and culture. We have previously proposed the *M*. *tb* DNA-synthetic enzyme thymidylate kinase (aka TMKmt) as an organism-specific growth and proliferation biomarker to reduce time-lines for detection of positive TB cultures. In this study, we explored the secretory levels of TMKmt in *M*. *tb* cultures and sputum, towards improving the overall laboratory diagnosis of TB.

**Methods and results:**

Modelling of TMKmt secretion in vitro was done by cloning, expressing and SDS-PAGE/MALDI-TOF detection of purified recombinant TMKmt in *E. coli*. TMKmt expression profiling in *M*. *tb* was done by qRT-PCR assay of related messenger ribonucleic acids (mRNA) and TMKmt antigen detection by Enzyme linked Immuno-absorbent Assay (EIA) among cultures of a pathogenic wild-type Ugandan strain (genotype 1) alongside the H37Rv laboratory strain. Owing to the high-load of pathogen in-culture, direct EIA on limiting dilutions of sputum were done to examine for assay sensitivity. A rise in TMKmt antigen levels was observed at 3 h post-innoculation among in vitro cultures of *E. coli*. The 1st of several cyclic spikes in TMKmt mRNA and antigen levels were detected at 2.5 h among in vitro cultures of the pathogenic wild-type Ugandan isolate alongside the laboratory *M*. *tb* strain. TMKmt antigen was detected up to between 1 × 10^−4^–1 × 10^−5^ (containing 10 and 1 CFUs/ml) dilutions of a microscopically designated 1+ (est. Acid Fast Bacillary load of 1 × 10^5^) patient sample.

**Conclusions:**

Detection of TMKmt expressed mRNA and Ag offers us opportune for instant diagnosis of *M*. *tb* in sputum, and reduction of timelines for positive pathogen detection in cultures to within 3 h.

**Electronic supplementary material:**

The online version of this article (doi:10.1186/s13104-017-2649-y) contains supplementary material, which is available to authorized users.

## Background

Tuberculosis (TB) remains a leading cause of human morbidity and mortality world-over. TB is caused by infection with *Mycobacterium tuberculosis* (*M*. *tb*) [1, 2]. Human infection with *M*. *tb* may either be latent *M*. *tb* (also designated latent *M*. *tb* infection, LTBI) or active (a.k.a active *M*. *tb* infection, ATBI) [2]. An approximate one-third of the global human population is thought to have LTBI. Each year, 8–10 million people develop ATBI, 2 million of whom die from TB related illnesses [1, 2].

Laboratory diagnosis of TB is traditionally based on microscopy and or culture. *On one hand,* confirmatory diagnosis of TB in the laboratory is based on culturing the organism on the traditional Lowenstein Jensen (LJ) or other new liquid medium. *M*. *tb* growth is slow, demanding several weeks to isolate and identify the organism in culture and thereby delaying timely diagnosis and subsequent patient management [3]. Traditional cultures of *M*. *tb* on LJ medium take weeks to read—thereby delaying the critical decision whether or not, to treat. Such delays in diagnosis and initiation of chemotherapy do not only increase morbidity and mortality from TB, but they also increase the risk of transmission in the community [4]. Of recent there has been introduction of molecular methods like GeneXpert^®^ MTB/RIF assays which detect *M*. *tb* quite fast. However, these assays are incapable of detecting live organisms, and do not replace the need for smear with microscopy for acid-fast bacilli, culture for mycobacteria, and growth-based drug susceptibility testing. Specifically, although application of these nucleic acids amplification tests (NAATS) either directly on sputum or cultures can increase the sensitivity for TB diagnosis among those with HIV co-infection as well as and reduce time-lines for positive culture detection, the need for microscopy or culture remains [3]. *On the other hand,* the use of microscopy for TB detection—whether conventional or inverted fluorescent, is only sensitive for a specified degree of bacillary load often not present in HIV co-infected persons. Moreover, studies evaluating TB treatment outcome have revealed that both sputum smear microscopy and culture have low positive predictive value [5]. Previous efforts to reduce timelines for *M*. *tb* culture have been based on radio-labelled carbon, acridinium-ester labelled DNA probes and other nucleic acids amplification tests (NAATS) [6–8]. These assays are nonethless, laborous to precisely undertake or replicate, and most take over 5 days to yield positive result. The low sensitivity of several immuno-assays for detecting *M*. *tb* in sputum at low bacillary loads (as is the case seen in HIV co-infection), has relegated several potentially emerging assays such as the Inducible Protein of 10Kda(IP-10), lipoarabinomannan (LAM) antigen and Adenosine-deaminase (ADA), and protein MPT64 (Capilia^®^) test to use strictly for purposes of reducing timelines of positive cultures [9–13]. Specifically, direct nucleic acid amplification tests (NAATs), while known to reduce the turnaround time for the laboratory diagnosis of tuberculosis by at least 2–4 days compared to conventional growth detection; have poor accuracy for *M*. *tb* due to low sensitivity and inhibition [14, 15]. Further more, because DNA/RNA probe assays—even when including an amplification step as is the case for the simultaneous amplification and testing (SAT) TB assay, are not sensitive enough [15]. Moreover, *DNA/RNA* probe assays require more time, manipulation, contamination controls, and costly reagents and instruments [7]. The available lateral flow based immune assay (e.g. Capilia^®^) has a detection limit of approximately 10^5^ CFU/ml, thereby requiring *M*. *tb* growth on solid or liquid medium prior to testing [12, 13]. In addition, Capillia^®^ tests tend to have a less than 100% sensitivity due to mutations in the MTP64 gene found by DNA sequencing in some isolates identified by conventional methods as belonging to the *M*. *tb* but negative in the lateral flow assays [13].

Our group has previously proposed the *M*. *tb* DNA-synthetic enzyme thymidylate kinase (aka TMKmt) as a specific organismal growth and proliferation biomarker to reduce time-lines for detection of positive TB cultures [16]. In this study, we explored the secretory levels of TMKmt in *M*. *tb* cultures and sputum, towards improving the overall laboratory diagnosis of TB.

## Results

### Modelling of TMKmt secretion in vitro using purified recombinant TMKmt cloned and expressed in *E. coli*

**Fig. 1:**
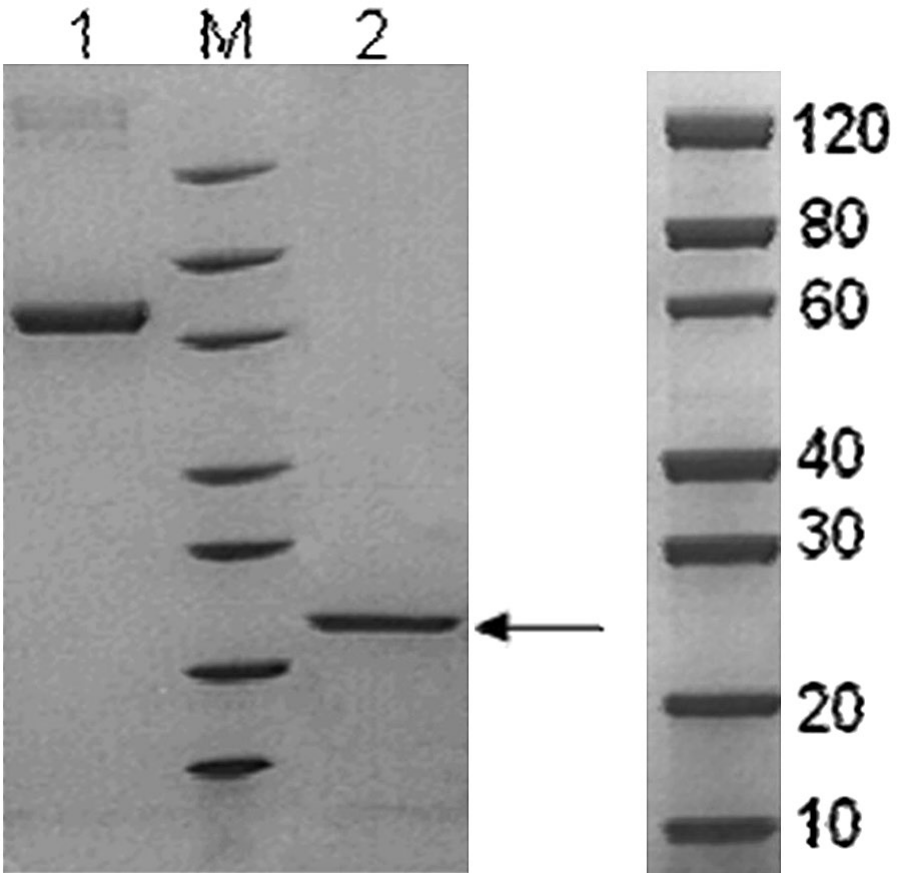
SDS-PAGE analysis of purified recombinant TMKmt cloned and expressed in *E. coli* BL21 (DE). This figures shows sodium dodecyl sulphate (SDS) Poly-Acrylamide Gel (PAGE) electrophoresis analysis of pooled fractions of urea dissolved precipatates from centrifugal *E. coli* cell-lysates filtered through a 0.22 μM sterilizing filter. SDS-PAGE was run on a 4–20% gel, followed by Coomassie blue starining. *Lane 1* is BSA (2.00 μg) while *Lane 2* is rTMKmt (2.00 μg). Protein Marker M is GenScript product Cat. # M00516

**Fig. 2:**
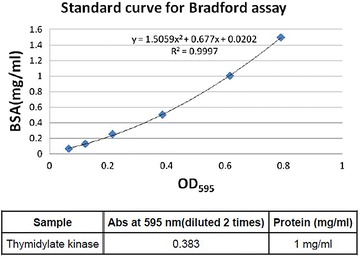
Bradford assay determination of concentration of recombinant TMKmt expressed in *E. coli* BL21 (DE). This figure shows the Bradford assay determination of concentration of recombinant TMKmt expressed in *E. coli* BL21 (DE). The concentration of expressed TMKmt determined by the Bradford assay was 1 mg/ml with an absorbance at 595 nm of 0.383

**Fig. 3:**
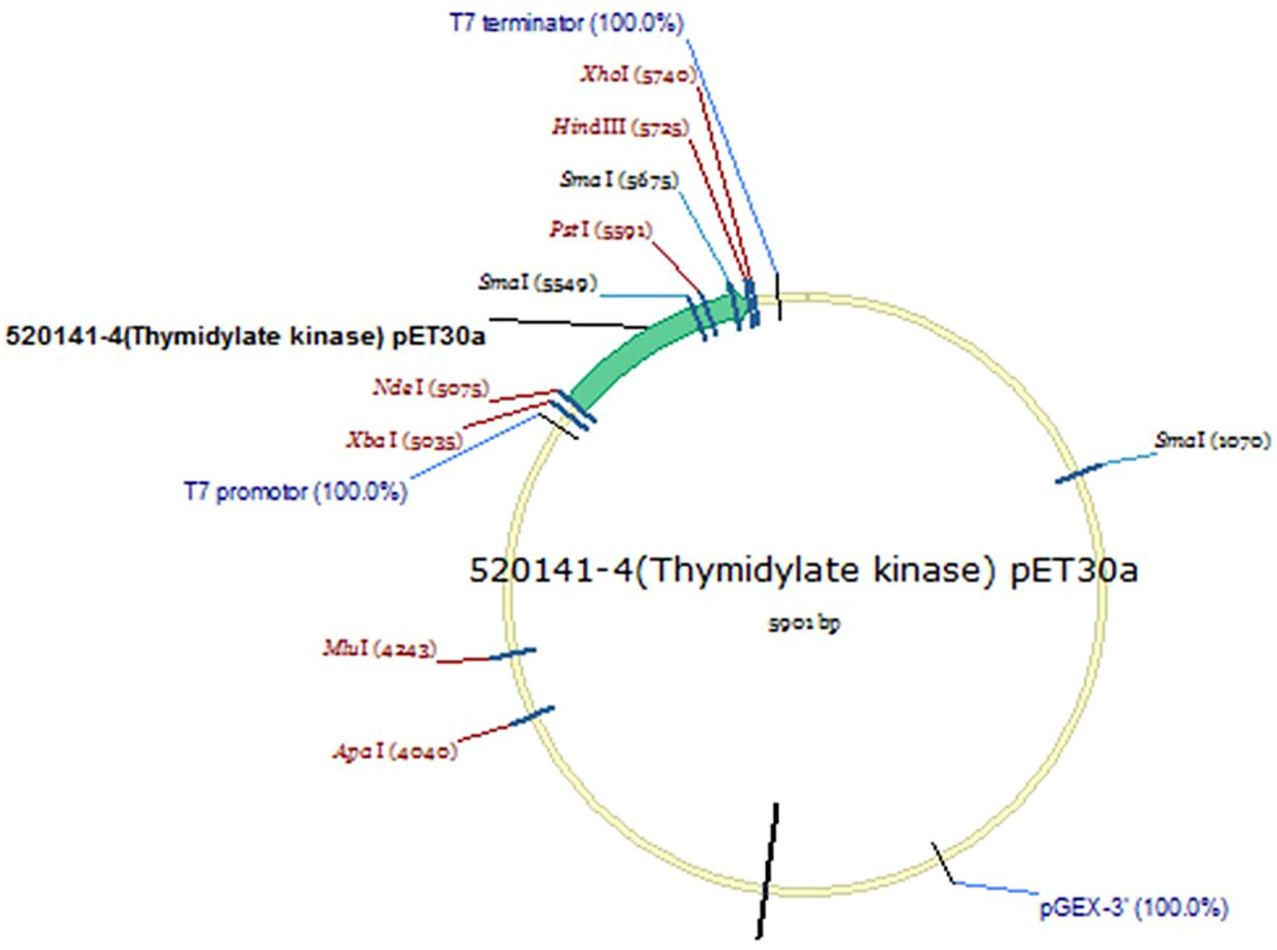
Construct of the pET30a construct used to clone and express TMKmt in *E. coli* Bl21 (DE). This figures shows the construct of the pET30a construct used to clone and express TMKmt in *E. coli* Bl21 (DE)

**Fig. 4:**
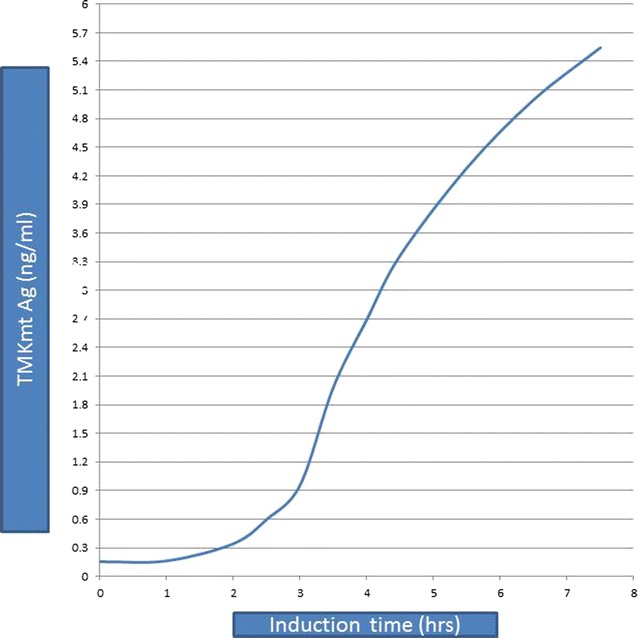
Expression profile of recombinant TMKmt Antigen in cultures of *E. coli* BL21 (DE) in vitro. This figure shows the expression profile of recombinant TMKmt Ag in cultures of *E. coli* BL21 (DE) in vitro. A rise in TMKmt antigen levels was observed by MALDI-TOFF analysis at 3 h post-innoculation among in vitro cultures of a *E. coli* BL21 (DE) transformed with pET30a plasmid vector transducing the TMKmt gene. Not the sigmoid pattern comprising an initial lag phase, intermediate log (or exponential) phase and a late stationery phase

In order to model the in vitro expression of the target *M*. *tb* growth and proliferation dependent antigen or biomarker (TMKmt), sodium dodecyl sulphate (SDS) Poly-Acrylamide Gel Electrophoresis (PAGE) and MALDI-TOF analysis were done on pooled fractions of urea dissolved precipatates from centrifugal cell-lysates of clones of *E. coli* filtered through a 0.22 uM sterilizing filter revealed that expressed rTMKmt has a molecular weight of 22, 635 and Isoelectric point of 7.66 (see Fig. 1). The concentration of expressed rTMKmt determined by the Bradford assay was 1 mg/ml with an absorbance at 595 nm of 0.383 (see Fig. 2). A rise in rTMKmt antigen levels was observed by MALDI-TOF at 3 h post-innoculation among in vitro cultures of the *E. coli* BL21 (DE) strain transformed with pET30a plasmid vector transducing the rTMKmt gene. The pET30a plasmid construct is shown in Fig. 3. TMKmt Ag expression profile in the same *E. coli* cultures is shown in Fig. 4. Details of recombinant TMKmt cloning, expression and purification in *E. coli* are shown in Additional file 1. Details of the expression profile of recombinant TMKmt (rTMKmt) in *E. coli* are shown in Additional file 2. These data demonstrate that recombinant TMKmt Ag expression follows the same sigmoid curve pattern of *E. coli* BL21 (DE) growth and proliferation in vitro. Recombinant TMKmt Ag expression in *E. coli* BL21 (DE) is characterized by an initial lag phase, followed by an exponential or log phase, and possibly a stationary phase. This is consistent with our earlier modelling of native TMKmt expression in *M*. *tb* and its potentail for reducing timelines for the diagnosis of positive TB cultures in vitro [16]. Specifically, recombinant TMKmt expression profiles in-culture preceed and are predictive of the growtth and proliferation of *E. coli* BL21 (DE) in vitro (see Figs. 2, 4, respectively).

### TMKmt mRNA and antigen expression profile among cultures of a pathogenic wild-type Ugandan isolate alongside a laboratory *M*. *tb* strain

**Fig. 5:**
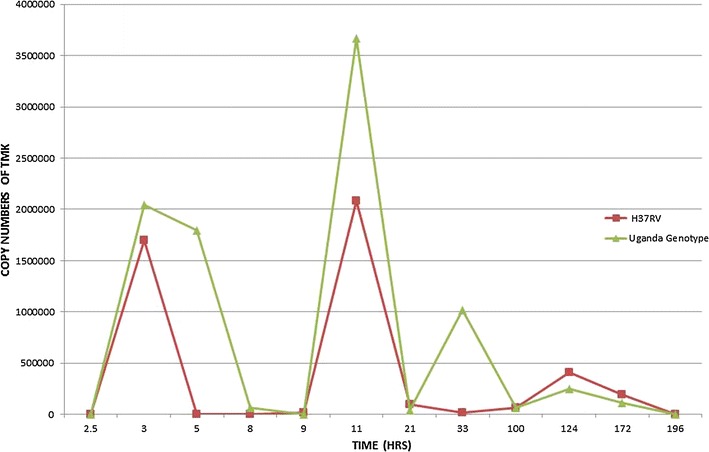
TMKmt mRNA expression profile among pure cultures of Uganda genotype 1 and H37Rv. This figure shows the TMKmt mRNA expression profile among pure cultures of Uganda genotype 1 and H37Rv. The 1st of several spikes in TMKmt rRNA levels was detected at 2.5 h (reaching its peak at 3 h) among in vitro cultures pathogenic wild-type Ugandan isolate alongside a laboratory *M*. *tb* strain The cyclic pattern expression of TMKmt Ag among H37RV, was different from the Ugandan genotype 1. Specifically, H37RV demonstrated an early but short lived spike in TMKmt Ag levels between 0 and 3 h post innoculation, with the next spike emerging at 12 h. This early spike in H37RV TMKmt Ag expression profiles, may explain why this lab strain grows faster than pathogenic strains (see Additional file 5: Figure S3). More important, *however*, is that regardless of these differences in cyclic patterns of TMKmt Ag expression, the Ugandan genotype displayed higher amplitudes of TMKmt mRNA expression. Although the 1st spike of TMKmt mRNA expression coincided with the 1st spike of TMKmt Ag levels, unlike the case for mRNA, TMKmt Ag expression profiles were followed by increasing lags between subsequent spikes relative to the 1st spike

**Fig. 6:**
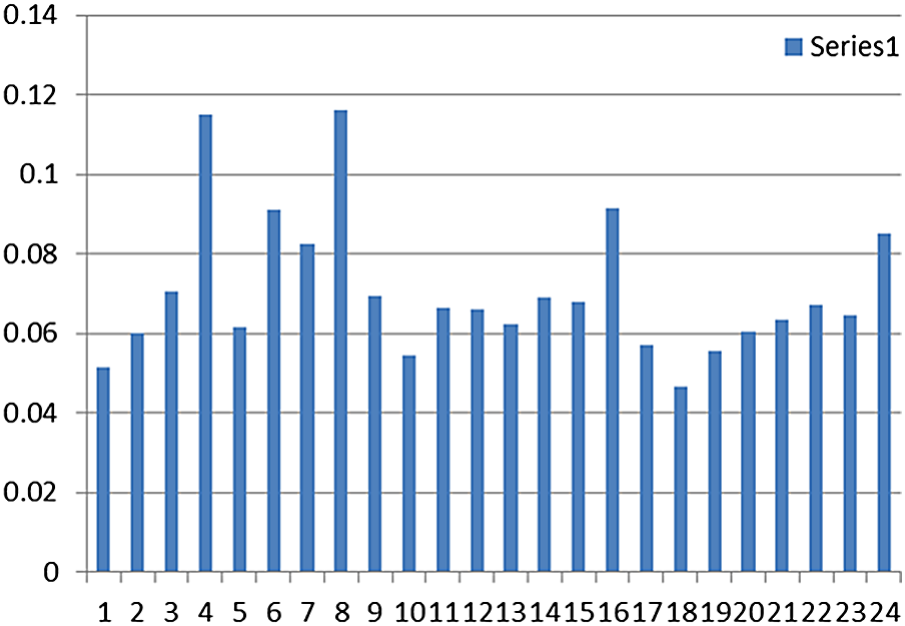
TMKmt Antigen expression profile among pure cultures of Uganda genotype 1. This figure shows the TMKmt antigen expression profile among pure cultures of Uganda genotype 1. In comparison, the cyclic pattern for expression of TMKmt Ag among H37RV (not shown), was different from the Ugandan genotype 1. Specifically, H37RV demonstrated an early but short lived spike in TMKmt Ag levels between 0 and 3 h post innoculation, with the next spike emerging at 12 h. This early spike in H37RV TMKmt Ag expression profiles, may explain why this lab strain grows faster than pathogenic strains. Important to note is that, regardless of these differences in cyclic patterns of TMKmt Ag expression, the Ugandan genotype displayed higher amplitudes of TMKmt mRNA expression

**Fig. 7:**
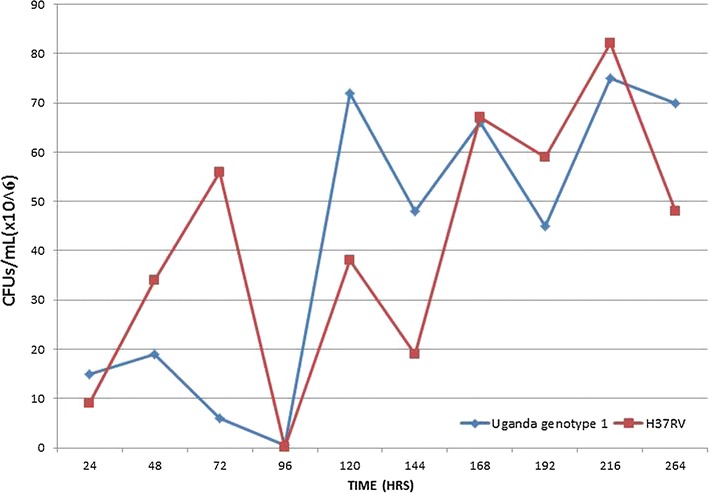
Colony forming units of in vitro cultures of Uganda genotype 1 and H37Rv. This figure shows *colony forming units* (*CFUs/ml*) *of in vitro* cultures of Uganda genotype 1 and H37Rv. At the point of 1st TMKmt mRNA and Ag spikes, CFU/ml of mycobacterium were below 1 × 10^10^

Unlike the sigmoid expression pattern observed with recombinant TMKmt expressed in *E. coli* observed above, TMKmt mRNA and antigen (Ag) displayed fluctuating spikes for cultures of both the Ugandan genotype 1 and H37RV. The 1st of several spikes in TMKmt mRNA levels was detected at 2.5 h (reaching its peak at 3 h) among in vitro cultures of pathogenic wild-type Ugandan isolate alongside the H37RV laboratory *M*. *tb* strain (see Fig. 5). This coincided with the 1st spike of TMKmt Ag levels, but unlike the case for mRNA, TMKmt Ag expression profiles were followed by increasing lags between subsequent spikes relative to the 1st spike (see Fig. 6). Details of Optimization of qRT-PCR of TMKmt and *M*. *tb* Pol1A are shown in Additional file 3: Figure S1. Additional file 4: Figure S2 shows gel electrophoresis of PCR amplicons of TMKmt and Pol1A. At the point of 1st TMKmt mRNA and Ag spikes, CFU/ml of mycobacterium were below 1 × 10^10^ (see Fig. 7). The spiking pattern of TMKmt mRNA and Ag expression may explain the slow growth of *M*. *tb*. Moreover, that spikes of TMKmt Ag are observed over increasingly time-lags from the 1st spike, suggest that TMKmt expression is cyclic and responds to chorum sensing in the micro-environment. The cyclic pattern expression of TMKmt Ag among H37RV, was different from the Ugandan genotype 1. Specifically, H37RV demonstrated an early on-set but short lived spike in TMKmt Ag levels between 0 and 3 h post innoculation, with the next spike emerging at 12 h. This early spike in H37RV TMKmt Ag expression profiles, may explain why this lab strain grows faster than pathogenic strains (see Additional file 5 Figure S3). More important, *however*, is that regardless of these differences in cyclic patterns of TMKmt Ag expression, the Ugandan genotype displayed higher amplitudes of TMKmt mRNA expression [best fit = 0.0775 ± 0.0057; 95% CI 0.0660–0.0889] relative to H37Rv[best fit = 0.0553 ± 0.0037; 95% CI 0.0478–0.0629] (see Table 1). Details of TMKmt Ag expression among the Ugandan genotype 1 and H37RV, are shown in Additional file 6: Figure S4 and Additional file 7.

**Table 1:** Statistical values for TMKmt Ag expression among serial samplings from cultures of Ugandan genotype 1 and H37Rv

Statistical parameter	Ugandan genotype	H37Rv
Best-fit values		
Slope	−0.0006 ± 0.0004	0.0002 ± 0.0003
Y-intercept when X = 0.0	0.07745 ± 0.0057	0.05526 ± 0.0037
X-intercept when Y = 0.0	141.9	−289.2
1/slope	−1833	5233
95% confidence intervals		
Slope	−0.0014 to 0.0003	−0.0003 to 0.0007
Y-intercept when X = 0.0	0.0660 to 0.0889	0.0478 to 0.0628
X-intercept when Y = 0.0	64.32 to +infinity	−infinity to −67.61
Goodness of fit		
r^2^	0.0392	0.0116
Sy.x	0.0191	0.0125
Is slope significantly non-zero?		
F	1.874	0.5386
DFn, DFd	1.000, 46.00	1.000, 46.00
P value	0.1776	0.4667
Deviation from zero?	Not significant	Not significant
Data		
Number of X values	24	24
Maximum number of Y replicates	2	2
Total number of values	48	48
Number of missing values	0	0

### Limit of detection of positive *M*. *tb* among serial dilutions of a 1+ acid fast bacilli patient sputum sample

**Fig. 8:**
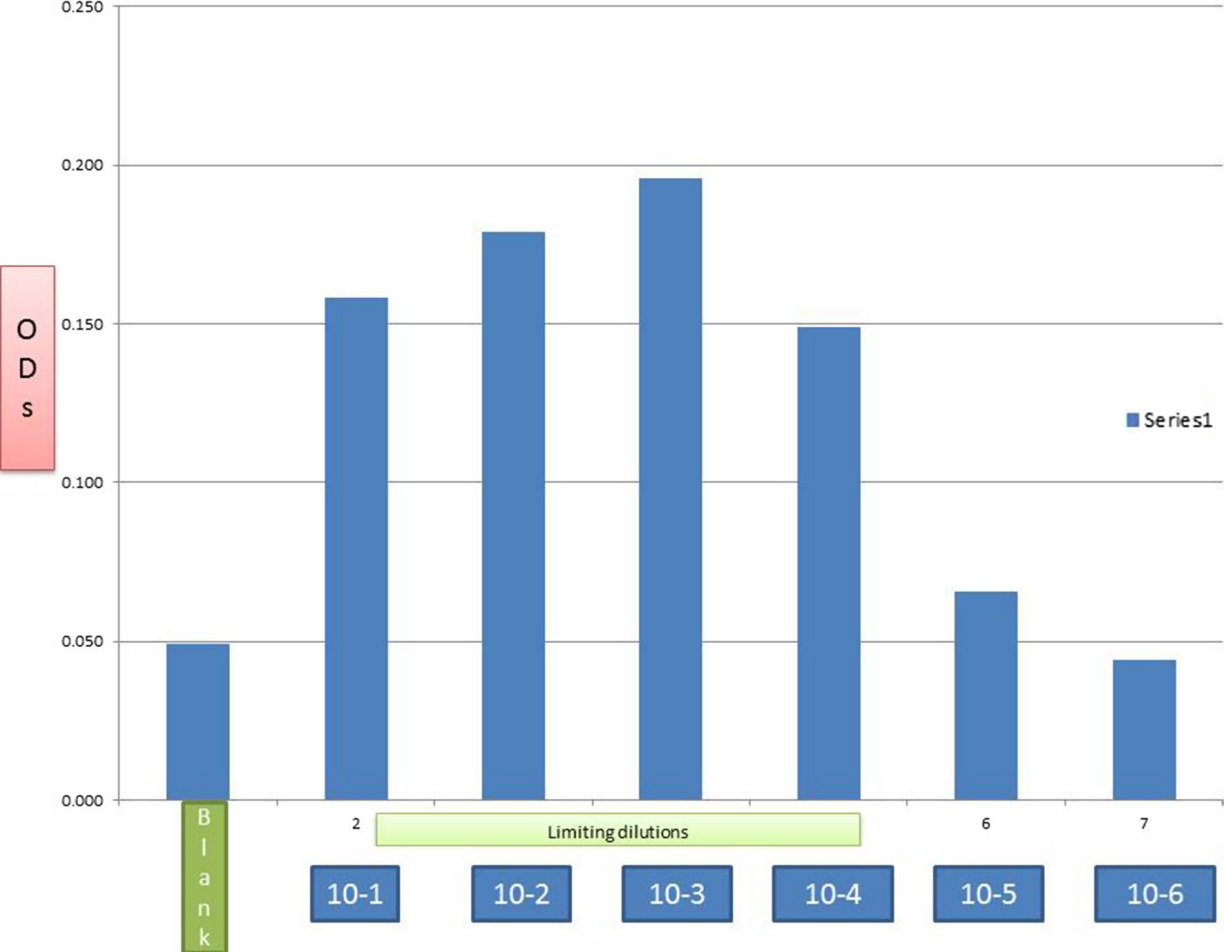
TMKmt Ag levels in serial dilutions of 1 + acid fast bacilli ladden patient sputum sample detected. This figure shows TMKmt Ag levels in serial dilutions of 1 + acid fast bacilli ladden patient sputum sample detected by PAb-0655. Direct Enzyme Immuno-assay (EIA) detection of TMKmt Ag using two custom polyclonal antibodies (PAb-0655 and PAb-0656) in the 1 × 10^−1^, 1 × 10^−2^, 1 × 10^−3^, 1 × 10^−4^, 1 × 10^−5^, 1 × 10^−6^ dilutions of sputum containing (considering the initial 1+ AFB sputum with 10^5^ CFUs/ml) approximately 10^4^, 10^3^, 10^2^, 10, 1, and 0.1 CFUs/ml of acid fast bacilli. Specifically, TMKmt antigen was detected up to 1 × 10^−4^ to 1 × 10^−5^ (containing 10 and 1 CFUs/ml) dilutions of a bead-treated, microscopically designated 1+ (est. Acid Fast Bacillary load of 1 × 10^5^) patient sample by PAb-0655 and PAb-0656, respectively

Considering the bacillary-load detection limit of approximately 10^5^ CFU/ml for the Capilia^®^ lateral flow immune assay, we set out to determine the detection limit of TMKmt for *M*. *tb* positivity in serial dilutions of a 1+ acid fast bacilli patient sputum sample (containing an estimated 1 × 10^5^ CFU/ml) [12, 13]. Theoretically, this work also evaluated the lung micro-environment as an in vivo *M*. *tb* culture system, thereby allowing for direct TMKmt antigen detection in unprocessed, un-cultured spot-sputum. Results for direct enzyme immuno-assay (EIA) detection of TMKmt Ag using two custom polyclonal antibodies (PAb-0655 and PAb-0656) in the 1 × 10^−1^, 1 × 10^−2^, 1 × 10^−3^, 1 × 10^−4^, 1 × 10^−5^, 1 × 10^−6^ dilutions of sputum containing (considering the initial 1+ AFB sputum with 10^5^ CFUs/ml) approximately 10^4^, 10^3^, 10^2^, 10, 1, and 0.1 CFUs/ml of acid fast bacilli are respectively shown in Fig. 8 and Additional file 8: Figure S5. GraphPad combined details of TMKmt Ag detection in the Uganda genotype 1 and H37Rv strains are shown in Additional file 9: Figure S6. Specifically, TMKmt antigen was detected up to 1 × 10^−4^–1 × 10^−5^ (containing 10 and 1 CFUs/ml) dilutions of a bead-treated, microscopically designated 1+ (est. Acid Fast Bacillary load of 1 × 10^5^) patient sample by PAb-0655 and PAb-0656, respectively. Statistical values for these results derived by the Prism software are shown in Table 2. The excel master file containing raw and adjusted ODs for TMKmt Ag detection in each serial dilutions is shown in Additional file 10. Additional file 9: Figure S6 depicts a GraphPad comparison of the variation of TMKmt in each serial dilution as detected by either PAb-0655 or PAb-0656.

**Table 2:** Statistical values for TMKmt Ag detection in serial dilutions of a 1 + AFB sputum sample

Statistiacl parameter	TMKmt Ag by PAb 0655	TMKmt Ag by PAb 0656
Best-fit values		
Slope	−0.0083 ± 0.0070	−0.0110 ± 0.0051
Y-intercept when X = 0.0	0.1532 ± 0.0311	0.1706 ± 0.0229
X-intercept when Y = 0.0	18.55	17.69
1/slope	−121.0	−103.7
95% confidence intervals		
Slope	−0.0228 to 0.0063	−0.0204 to 0.0011
Y-intercept when X = 0.0	0.0882 to 0.2183	0.1227 to 0.2184
X-intercept when Y = 0.0	8.519 to +infinity	9.999 to +infinity
Goodness of fit		
r^2^	0.06918	0.1576
Sy.x	0.06372	0.04688
Is slope significantly non-zero?		
F	1.412	3.554
DFn, DFd	1.000, 19.00	1.000, 19.00
P value	0.2494	0.0748
Deviation from zero?	Not significant	Not significant
Data		
Number of X values	7	7
Maximum number of Y replicates	3	3
Total number of values	21	21
Number of missing values	0	0

## Discussion

We present experimental data that supports the view that detection of TMKmt expressed mRNA and Ag offers us opportune for instant diagnosis of *M*. *tb* in sputum, and reduction of timelines for positive pathogen detection in cultures to within 3 h. These results provide basis for not only designing rapid diagnostic tests (RDT) for TB detection at the point of care (POC) in sputum and possibly other bodily fluids, but also reducing time-lines for revealing positive TB cultures. These are critical bottle-necks in the public health control of TB, in that they address the challenge of cost, ease-of technological uptake, and turn-around times for TB diagnosis [1, 2].

*First*, we show that recombinant TMKmt (rTMKmt) mRNA and Ag expression in *E. coli* BL21 (DE) is respectively detectable on qRT-PCR and ELISA platforms within 3 h of sub-culturing (see Figs. 1, 2, 3. Figure 4; Additional file 1, 2). This is consistent with our earlier modelling of native TMKmt expression in *M*. *tb* and its potential for reducing timelines for the diagnosis of positive TB cultures in vitro [16]. *Second,* although expression of native TMKmt mRNA and Ag among the wild-type Ugandan genotype and H37RV laboratory strain of *M*. *tb* did not display the classical sigmoid curve of recombinant TMKmt expression predictive of the in vitro growth of *E. coli* BL21 (DE) (see Figs. 5, 6, 7; Additional file 3: Figure S1, Additional file 4: Figure S2, Additional file 5: Figure S3, Additional file 6: Figure S4, Additional file 7); the rise in the amplitudes of each subsequent spikes could potentially be used to predict *M*. *tb* growth and proliferation within 2.5 h (mRNA) and 3 h (Ag) respectively. This is an overwhelmingly significant reduction in the 1–5 day time-lines for detection of positive *M*. *tb* cultures by the current immuno, radio-labelled or NAATs (GenXpert^®^) methods on market [2, 3, 6, 9, 10, 12, 13]. Moreover, that drug sensitivity testing (DST) for *M*. *tb* is largely based on cultures, these results are in-line with and support our previous theoretical predictions to apply TMKmt detection towards reducing time-lines for TB DST [16]. This data may suffice to indirectly encourage investments in parallel research and development (R &) of antagonists of TMKmt as drug targets [17–20]. *Third*, considering the bacillary-load detection limit of approximately 10^5^ CFU/ml for the Capilia^®^ lateral flow immune assay, we set out to determine the limit of detection for TMKmt Ag conoistent with *M*. *tb* positivity in serial dilutions of a 1+ acid fast bacilli patient sputum sample (containing an estimated 1 × 10^5^ CFU/ml) [12, 13]. TMKmt antigen was detected up to 1 × 10^−4^–1 × 10^−5^ (containing 10 and 1 CFUs/ml) dilutions of a bead-treated, microscopically designated 1+ (est. Acid Fast Bacillary load of 1 × 10^5^) patient sample by PAb-0655 and PAb-0656, respectively (see Fig. 8; Additional file 6: Figure S4, Additional file 8: Figure S5, Additional file 10; alongside Table 2). These data demonstrate that direct detection of TMKmt Ag in sputum has a superior detection-limit of 10 and 1 CFUs/ml compared to that of e Capilia^®^ lateral flow immune assay (10^5^ CFU/ml) [12, 13]. Therefore, TMKmt Ag detection is a viable point of care immune assay to evaluate towards direct detection of *M*. *tb* positivity in sputum of patients with pulmonary TB (PTB). Moreover, the assay may theoretically be applicable to other TB specimen, including ascites (abdominal TB), pleural fluid (PTB), cerebral spinal fluid (CSF, TB meningitis) and other solid tissue specimen.

The translational significance of our results is two fold: (1) that rapid diagnostic tests (RDTs) for both TMKmt Ag and or Ab based on the micro-electro mechanical systems (MEMS) microfluidic or lateral flow immunochromatographic strip test (LFT) platforms can be used to instantaneously designate TB disease in sputum and possibly other bodily fluids such as ascites, cerebral spinal fluid, pleural effusions and lymphnode aspirates (a recent report has affirmed the ability to sero-diagnose TB among PLWHA on basis of TMKmt Ag and Ab detection EIAs done using patient blood [21]), and (2) that TMKmt mRNA or Ag detection in-culture using either microfluidic MEMS (exploiting labelled probes say forster resonance energy transfer, FRET) or LFT-RDTs respectively, can designate positive cultures within 3 h—thereby allowing the health-care workers to decide on whether or not to treat, while the patient waits within an out-patient setting. Moreover, both these can be adopted to design culture independent, rapid assays for phenotyping TB drug susceptibility and or resistance as previously anticipated [16].

Deploying such an assay is predicted to overcome challenges for TB diagnosis in many TB high-burden resource limited settings, by overcoming delays for diagnosis and allowing for timely initiation of chemotherapy, as well as easying monitoring for treatment efficacy and predicting outcome or resistance [4, 5, 11]. A potential source of false positive is the distant homology of TMKmt antigens used to TMK of other non-MTB bacteria (see Additional file 11). Specifically, potential for cross-reactivity with TMK of other bacteria species other than TMKmt as predicted by basic local sequence alignments tool (BLAST) analysis across the National Center for Biotechnology Information (NCBI) microbial genome database. Although 100% sequence identity was only observed with TMKmt from various *M*. *tb* species, additional protein homology of more than 60% sequence identity was noted with TMK of Corynebacteria spp., Gordonia spp., Tomitella spp., Rhodococcus spp., Segniliparus spp., Dietzila spp., and Tulicella spp. Notable, however, is that the latter are not documented common pathogens of opportunistic co-infection with HIV/AIDS as the case with *M*. *tb* [1, 2].

## Conclusions

In conclusion, detection of TMKmt expressed mRNA and Ag offers us opportune for instant diagnosis of *M*. *tb* in sputum, and reduction of timelines related positive pathogen cultures to within 3 h. This could overcome the prevailing diagnostic delays at the point of care within TB high-burden resource limited settings, and improve treatment outcome by enabling instant treatment-monitoring and drug sensitivity testing.

## Methods

### Pre-amble to methodology

The identity of the two TMKmt epitopes used to derive polyclonal antibodies (PAb-0655 and PAb-0656) used for TMKmt antigen (Ag) detection enzyme immuno-assays (EIAs) in this study, has been described elsewhere [22, 23]. *Briefly*, (1) using the entire 214 amino acids sequences of TMKmt (SP “|O05891|”) and five biophysical profiles (accessibility, antigenicity, beta-turn, flexibility, and hydrophilicity) in the immune epitope database analysis resource (IEDB-AR), the 27 AA long 148_ERSRGRAQRDPGRARDNYERDAELQQR peptide was predicted to be the best continuous linear B cell epitope by all profiles (4/5, 80%) except antigenicity[24–28]. *Second*, using the crystal structure of TMKmt (PdB entry: “1g3u”) and discontinuous B cell epitope software DiscoTope, we derived 22 amino acids (A:G57, A:E148, A:S150, A:R151, A:G152, A:R153, A:A154, A:Q155, A:R156, A:D157, A:P158, A:G159, A:A160, A:A161, A:R162, A:A163, A:N164, A:E166, A:R167, A:D168, A:A169, A:T179) as the best discontinuous or non-linear epitope [26]. Both peptide-epitope sequences were cross validated by BLAST across microbial- and conserved domain databases (CDD) [28, 29] (see Additional file 11). These two synthetic TMKmt-peptide-epitopes were used to derive the two rabbit TMKmt specific polyclonal antibodies (PAb-0655 and PAb-0656 respectively, GeneCUST, Luxemburg) used to detected TMKmt Antigen (Ag) levels in purified *M*. *tb* cultures and sputum dilutions.

### Modelling of TMKmt secretion in vitro using purified recombinant TMKmt cloned and expressed in *E. coli*

The full length gene of TMKmt (NCBI Gene ID|71609|” was designed with related tags for each construct to facilitate purification. The complete sequence was subcloned between NdeI (5074) and *Hin*dIII (5735) restriction sites of a pET30a plasmid. A T7 promotor was placed between the upstream of the *Nde*I(5074) and a *Xba*I[5035] restriction sites; while a T7 terminator was placed downstream of the *Xh0*I (5740) and a *Xh0*I(5740) restriction site. An established purified culture of *E. coli* BL21 (DE3) was transformed with the recombinant TMKmt gene-carrying pET30a plasmid. A single colony was inoculated into medium ampicillin and kanamycin. Cultures were incubated at 37 °C at 200 rpm. IPTG was introduced for induction. SDS-PAGE was used to monitor the expression. Samplings were isolated at 1.0, 2.0, 2.5, 3.0, 3.5, 4.0, 4.5, 5.0, 5.5, 6.0, 6.5, 7.0 and 7.5 h; and cell pellets isolated by centrifugation. Cell pellets were lysed by sonication, and precipitate after centrifugation was dissolved in urea. Fractions were pooled and refolded followed by 0.22 uM filter sterilization. Proteins were analysed by MALDI-TOF and SDS-PAGE by using standard protocols for molecular weight and purity measurements. The concentration was determined by Bradford assay with Bovine serum albumin (BSA) as a standard (see Additional file 1 for details).

### TMKmt mRNA and antigen expression profile among cultures of a pathogenic wild-type Ugandan isolate alongside a laboratory *M*. *tb* strain

Isolates of the Ugandan genotype 1 used in this study were retrieved from the Mycobacteriology Level III facility at MakCHS, Kampala, Uganda. There were around seventy-seven samples where the genotype of interest (Uganda genotype 1) was represented by more than six strains. Thus six representative strains for the genotype were retrieved, thawed and inoculated onto Middlebrook 7H10 (Difco™Becton, Dickson and Company, Sparks, MD, USA) solid media. Two strains that showed acceptable growth fitness patterns (at least showing colonies on the thirteenth day) and no contamination (this was the fitness test) were selected for use in the study. (a) Culture of Isolates: Isolates were thawed and individually streaked onto Middlebrook 7H10 and incubated in a Carbon dioxide incubator at 37 °C (ThermoScientific Forma, Reach in-CO_2_Incubator, USA) for up to 13 days. 10 ml of 2 Marcfland (6 × 10^8^ CFU) standard solution were prepared from each plate using sterile distilled water and subsequently emulsified in 200 ml of Middlebrook 7H9 (Difco™Becton, Dickson and Company, Sparks, MD, USA) with tween^R^80 (Sigma-Aldrich, Dussedat, Germany) supplemented with OADC(Sigma-Aldrich, Fluka-M0678 Dussedat, Germany). The culturing bottles were incubated at 37 °C in incubator shaker (New Brunswick™ Excella^R^ E24/E24R, Eppendorf, NY. USA). (b) Collection samples for mRNA assay: 0.2 ml aliquots were collected at time intervals of 2.5, 3, 5, 8, 11, 15, 21, 33, 100, 124, 172, and 196 h. The aliquots were collected in triplicates and stored in RNAprotect^R^ (Qiagen Technologies, Germany)—a stabilizing reagent for RNA and kept in a −80 °C freezer (Innova U101 NewBrunswick, Eppendorf, NY, USA) prior to total genomic RNA extraction. (c) Estimating the number of CFUs: Serial dilution, spread-plate technique was used to estimate the number of CFUs that could be recovered from each bottle each day as the broth was incubated throughout. 1 ml from each culture bottle each day was diluted fivefold in Middlebrook 7H9 and 0.1 ml of each dilution was plated on Middlebrook 710 solid media in triplicates. The CFUs were visually counted every after 14 days and recorded and the average was recorded. (d) Extracting total genomic mRNA and converting it into cDNA: The aliquots were thawed from the freezer, centrifuged and the cell pellet harvested from which total RNA was extracted using RNeasy^®^ Mini kit (Qiagen Technologies, USA) which was subsequently treated with DNase enzyme using RNase-Free DNase Set (Paxgene, http://www.PreAnalytiX.com). Afterwards the RNA samples were assessed for purity by measuring their absorbance at 260 and 280 nm in Tris EDTA buffer using NanoDrop1000 Spectrophotometer (Thermo Fisher Scientific, Wilmington, Delaware USA) and only samples with 260/280 ratio of approximately 2 were considered for downward assays. Eventually the RNA was converted to cDNA using ProtoScript^®^ First Strand cDNA Synthesis Kit (New England Biolabs, 240 County road. Ipswich, UK). (e) Running Real time PCR assays: Conditions for real time PCR assays were optimized in a conventional T100™ Thermocycler (Bio-rad laboratories, Hertfordshire HP2D7X0, UK) using genomic *M*. *tb* DNA with different dilutions employing in-house designed primers for amplification of a 329 bp product length of TMK*mt* gene (F and R respectively 5-GTTGGTGGAAAAGCTGTCCG-3 and 5-ATTCGATCCGCTGAACCCAG-3)., Additional file 4: Figure S2 shows gel electrophoresis of optimized PCR amplicons of TMKmt and Pol1A. The conditions for the PCR reaction were as follows: denaturation temperature was 95 °C for 5 min, additional denaturation step at 95 °C for 45 s, annealing temperature at 54 °C at 45 s and extension for 45 s at 72 °C and final extension at 72 °C for 10 min. The cycle was repeated 35 times. The amplification products were separated on a 1% agarose gel electrophoresis and analyzed using BioDoc-It^®^ Imaging System (UVP analytica company, Chicago, USA). The conditions were once again optimized on to Rotor Gene Q thermocycler (Qiagen Technologies, USA) for real time assay using 2xQuantitect SYBR green PCR mix (Qiagen Technologies, USA) instead of Taq2x master mix but keeping the rest of reagents plus their volumes as well as the reaction conditions constant. Details of Optimization of qRT-PCR of TMKmt and *M*. *tb* Pol1A are shown in Additional file 3: Figure: S1. Thus these were the volumes and conditions used in the subsequent assays. To improve on SYBR green quantification, a high temperature fluorescence measurement point at the end of fifth segment was performed, this melts the unspecific products below chosen temperature and thus eliminates non-specific fluorescence signals. Three negative controls were run for each assay and all samples that had cycle thresholds (CTs) above or equal to any of the negative controls were excluded from analysis. To control for expression, *M*. *tb* PolA 1 gene that code for DNA polymerase 1 was also assayed for its expression using the exact conditions as TMKmt but using in-house designed primers 5′-ACCCAAAGCCTTGCATGAG-3′ and 5′-ACCGGCACTTTCCATCTTC-3′ as forward and reverse respectively targeting a TMKmt amplicon of 382 bp. (f) TMKmt Ag detection enzyme immuno-assays: For detection of TMKmt Ag in frozen centrifugal supernatants of samplings from purified cultures of the Ugandan genotype 1 and H37Rv preserved in SDS, (1) several study aliquotes were prepared by dissolving 1 μl of each sampling in 1000 μl of freshly prepared phosphate buffered saline. (2) 100 µl of resultant serum-diluent was then pipetted into duplicate wells of a sterile 96-well microtiter plate (Nunc) and the plate incubated overnight. (3) The plated wells were then blocked once the following day using 5% BSA in PBS and incubated at 37 °C for 30 min, after which excess solution was discarded and plate left to dry. Blank wells were also made, by adding only PBS rather than sample. (4) TMKmt specific rabbit polyclonals (either PAb-0655 or PAb-0656) were added, and wells incubated at 37 °C for 30 min, after which excess solution was discarded and plate left to dry. The wells-in-use were then washed with PBS three times using an automated plate-washer. (5) 100 µls of goat anti-rabbit IgG horse-radish peroxidase conjugate was added, and the plates incubated at 37 °C for another 1 h. During this incubation, the enzyme–substrate was prepared by adding 1 volume of substrate (TMB) to 1 volume of diluent (hydrogen peroxide) in volumes enough for all thewells in use. (6) 200 µl of freshly prepared substrate was added to each well (purple-bluish color developed in all except A-BX1 blank wells). (7) The reaction was stopped by adding 100 µl of dilute (1 mol/l) H_2_SO_4_. The intensity of the reaction in each well was hence after determined by reading the plate at an optical density (OD) of 450 nm using a single filter of an automated ELISA plate reader (PR 3100, Bio-Rad).

### Limit of detection of positive *M*. *tb* among serial dilutions of a 1+ acid fast bacilli patient sputum sample

An intial volume 1 μl of a 1+ AFB patient sample was homogenously diluted (by steering on an automated shaker at 100 rpm) in 10 μls of phosphate buffered saline (PBS). Subsequently, 1 μl of this resultant 1 × 10^−1^ or 0.1 μl homogenous concentration of sputum was diluted in a fresh solution of 10 μls of PBS. The process was repeated five times to yield 1 × 10^−1^, 1 × 10^−2^, 1 × 10^−3^, 1 × 10^−4^, 1 × 10^−5^, 1 × 10^−6^ dilutions of sputum. These issuing serial dilutions of a 1+ AFB sputum with 10^5^ CFUs/ml are estimated to contain approximately 10^4^, 10^3^, 10^2^, 10, 1 and 0.1 CFUs/ml of acid fast bacilli. For detection of TMKmt Ag in serial dilutions of the 1+ AFB patient sputum sample (1) several study aliquotes were prepared by dissolving 1 µl of each sputum-dilution in 1000 µl of freshly prepared phosphate buffered saline. (2) 100 µl of resultant serum-diluent was then pipetted into triplicate wells of a sterile 96-well microtiter plate (Nunc) and the plate incubated overnight. (3) The plated wells were then blocked once the following day using 5% BSA in PBS and incubated at 37 °C for 30 min, after which excess solution was discarded and plate left to dry. Blank wells were also made, by adding only PBS rather than sample. (4) TMKmt specific rabbit polyclonals (either PAb-0655 or PAb-0656) incubated at 37 °C for 30 min, after which excess solution was discarded and plate left to dry. The wells-in-use were then washed with PBS three times using an automated plate-washer. (5) 100 µls of goat anti-rabbit IgG horse-radish peroxidase conjugate was added, and the plates incubated at 37 °C for another 1 h. During this incubation, the enzyme–substrate was prepared by adding 1 volume of substrate (TMB) to 1 volume of diluent (hydrogen peroxide) in volumes enough for all thewells in use. (6) 200 µl of freshly prepared substrate was added to each well (purple-bluish color developed in all except A-BX1 blank wells). (7) The reaction was stopped by adding 100 µl of dilute (1 mol/l) H_2_SO_4_. The intensity of the reaction in each well was hence after determined by reading the plate at an optical density (OD) of 450 nm using a single filter of an automated ELISA plate reader (PR 3100, Bio-Rad).

## Additional files

**Additional file 1.** This file offers details of recombinant TMKmt cloning, expression and purification in *E. coli* BL21 (DE).

**Additional file 2.** This file offers details of the expression profile of recombinant TMKmt in *E. coli* BL21 (DE).

**Additional file 3.** This figures shows optimization curves of qRT-PCR for TMKmt and M*.tb* Pol1A.

**Additional file 4.** This figures depicts gel electrophoris analysis of PCR amplicons of TMKmt and M.tb Pol1A. Note the 22kDA placement of the TMKmt amplicon relative to protein marker M.

**Additional file 5.** This figure shows TMKmt Ag levels in serial dilutions of 1+ acid fast baccili ladden patient sputum sample detected by PAb-0656. Direct Enzyme Immuno-assay (EIA) were conducted using two custom polyclonal antibodies (PAb-0655 and PAb-0656) in the 1x10-1, 1x10-2, 1x10-3, 1x10-4, 1x10-4, 1x10-5, 1x10-6 dilutions of sputum containing (considering the initial 1+ AFB sputum with 10^5^ CFUs/ml) approximately 10^4^, 10^3^, 10^2^, 10, 1, and 0.1 CFUs/ml of acid fast bacilli are respectively. Note that relative to the blank, TMKmt Ag was detected upto 1x10-4 to 1x10-5 (containing 10 and 1 CFUs/ml) dilutions of a microscopically designated 1+ (est. Acid Fast Bacillary load of 1x10^5^) patient sample by PAb-0655 and PAb-0656, respectively.

**Additional file 6.** This figure shows the GraphPad combined TMKmt antigen expression profile among pure cultures of Uganda genotype 1 detected by PAb-0655 and PAb-0656. In comparision, the cyclic pattern for expression of TMKmt Ag among H37RV (not shown), was different from the Ugandan genotype 1. Specifically, H37RV demonstrated an early but short lived spike in TMKmt Ag levels between 0 and 3 hours post innoculation, with the next spike emerging at 12 hours. This early spike in H37RV TMKmt Ag expression profiles, may explain why this lab strain grows faster than pathogenic strains. Important to note is that, regardless of these differences in cyclic patterns of TMKmt Ag expression, the Ugandan genotype displayed higher amplitudes of TMKmt mRNA expression.

**Additional file 7.** This file offers details of TMKmt Ag expression among the Ugandan genotype 1 and H37RV.

**Additional file 8.** This figure shows TMKmt Ag levels in serial dilutions of 1+ acid fast baccili ladden patient sputum sample detected by PAb-0656 conjugate. Direct Enzyme Immuno-assay (EIA) were conducted using PAb-0656 conjuagte in the 1x10-1, 1x10-2, 1x10-3, 1x10-4, 1x10-5, 1x10-6 dilutions of sputum containing (considering the initial 1+ AFB sputum with 10^5^ CFUs/ml) approximately 10^4^, 10^3^, 10^2^, 10, 1 and 0.1 CFUs/ml of acid fast bacilli are respectively. Note that relative to the blank, TMKmt Ag was detected upto 1x10-5 (containing 1 CFUs/ml) dilutions of a microscopically designated 1+ (est. Acid Fast Bacillary load of 1x105) patient sample by PAb-0656.

**Additional file 9.** This figure shows GraphPad combined TMKmt Ag levels in serial dilutions of 1+ acid fast baccili ladden patient sputum sample detected by PAb-0655 and PAb-0656 conjugates, respectively. Direct Enzyme Immuno-assay (EIA) were conducted using either PAb-0655 or PAb-0656 conjuagte in the 1x10-1, 1x10-2, 1x10-3, 1x10-4, 1x10-5, 1x10-6 dilutions of sputum containing (considering the initial 1+ AFB sputum with 105 CFUs/ml) approximately 10^4^, 10^3^, 10^2^, 10, 1, and 0.1 CFUs/ml of acid fast bacilli are respectively. Note that relative to the blank, TMKmt Ag was detected upto 1x10-4 and 1x10-5 (containing 10 and 1 CFUs/ml) dilutions of a microscopically designated 1+ (est. Acid Fast Bacillary load of 1x10^5^) patient sample by PAb-0655 and PAb-0656, respectively.

**Additional file 10.** This file offers details of raw and adjusted ODs for TMKmt Ag detection in each serial dilutions of a 1+ AFB patient sputum sample.

**Additional file 11.** This file offers details of cross validated by BLAST across the NCBI microbial- databases.

## Abbreviations

Ab: antibody
AFB: acid fast baccilli
Ag: antigen
OD: optical density
EIA: enzyme immuno-assays
ELISA: enzyme linked immuno-adsorbent assays
*M*. *tb*: *Mycobacterium tuberculosis*
TB: tuberculosis
TMKmt: *Mycobacterium tuberculosis* thymidylate kinase

## References

[1] Snider DEJ, Raviglione M, Kochi A, Bloom BR (1994). Global burden of tuberculosis. Tuberculosis.

[2] Wheeler PR, Ratledge C, Bloom BR (1996). Pathogenesis, protection, and control. Tuberculosis.

[3] Soini H, Musser JM (2001). Molecular diagnosis of mycobacteria. Clin Chem.

[4] Kiwuwa MS, Charles K, Mayanja-Kizza H (2005). Patient and health service delay in pulmonary tuberculosis patients attending a referral hospital: a cross-sectional study. BMC Public Health.

[5] Horne DJ, Royce SE, Gooze L, Narita M, Hopewell PC, Nahid P (2010). Sputum monitoring during tuberculosis treatment for predicting outcome: systematic review and meta-analysis. Lancet Infect Dis.

[6] Larsson L, Odham GR, Westerdahl G (1981). Use of selected ion monitoring for detection of tuberculostearic and mycocerosic acid in Mycobacterium and in Five day old cultures of sputum specimen from patients with pulmonary Tuberculosis. Acta Pathol Microbiol Scand Sect B Microbiol.

[7] Evans KD, Nakasone AS, Sutherland PA, de la Maza LM, Peterson EM (1992). Identification of *Mycobacterium tuberculosis* and *Mycobacterium avium*-M. intracellulare directly from primary BACTEC cultures by using acridinium-ester-labeled DNA probes. J Clin Microbiol.

[8] Flores LL, Pai M, Colford JM, Riley LW (2005). In-house nucleic acid amplification tests for the detection of *Mycobacterium tuberculosis* in sputum specimens: meta-analysis and Meta-regression. BMC Microbiol.

[9] Dheda K, Van-Zyl Smit RN, Sechi LA, Badri M, Meldau R, Symons G (1999). Clinical diagnostic utility of IP-10 and LAM antigen levels for the diagnosis of tuberculous pleural effusions in a high burden setting. PLoS ONE.

[10] Boehme C (2005). Detection of mycobacterial lipoarabinomannan with an antigen-capture ELISA in unprocessed urine of Tanzanian patients with suspected tuberculosis. Trans R Soc Trop Med Hyg.

[11] Getahun H, Harrington M, O’Brien R, Nunn P (2007). Diagnosis of smear-negative pulmonary tuberculosis in people with HIV infection or AIDS in resource-constrained settings: informing urgent policy changes. Lancet.

[12] Hillemann D, Rüsch-Gerdes S, Richter E (2005). Application of the Capilia TB assay for culture confirmation of *Mycobacterium tuberculosis* complex isolates [Short Communication]. Int J Tuberc Lung Dis.

[13] Nakamura RM, Velmonte MA, Kawajiri K, Ang CF, Frias RA, Mendoza MT (1998). MPB64 mycobacterial antigen: a new skin-test reagent through patch method for rapid diagnosis of active tuberculosis. Int J Tuberc Lung Dis.

[14] Mikhailovich V, Lapa S, Gryadunov D, Sobolev A, Strizhkov B, Chernyh N (2001). Identification of rifampin-resistant *Mycobacterium tuberculosis* strains by hybridization, PCR, and ligase detection reaction on oligonucleotide microchips. J Clin Microbiol.

[15] Parsons LM, Somosk AK, Gutierrez C, Lee E, Paramasivan CN, Abimiku AI (2012). Laboratory diagnosis of tuberculosis in resource-poor countries: challenges and opportunities. Clin Microbiol Rev.

[16] Wayengera M (2009). Theoretical basis for reducing time-lines to the designation of positive *Mycobacterium tuberculosis* cultures using thymidylate kinase (TMK) assays. Theor Biol Med Model..

[17] Munier-Lehmann H, Chaffotte A, Pochet S, Labesse G (2001). Thymidylate kinase of *Mycobacterium tuberculosis*: a chimera sharing properties common to eukaryotic and bacterial enzymes. Protein Sci.

[18] Fioravanti E, Adam V, Munier-Lehmann H, Bourgeois D (2005). The Crystal structure of *Mycobacterium tuberculosis* thymidylate kinase in complex with 3′-azidodeoxythymidine monophosphate suggests a mechanism for competitive inhibition. Biochemistry.

[19] de la Sierra IL, Munier-Lehmann H, Gilles AM, Bârzu O, Delarue M (2001). X-ray structure of TMP kinase from mycobacteria tuberculosis complexed with TMP at 1.95 A resolution. J Mol Biol.

[20] Familiar O, Munier-Lehmann H, Aínsa JA, Camarasa MJ, Pérez-Pérez MJ (2010). Design, synthesis and inhibitory activity against *Mycobacterium tuberculosis* thymidine monophosphate kinase of acyclic nucleoside analogues with a distal imidazoquinolinone. Eur J Med Chem.

[21] Wayengera M, Mwebaza I, Welishe J, Nakimuli C, Kateete DP (2017). Sero-diagnosis of active *Mycobacterium tuberculosis* disease among HIV co-infected persons using thymidylate kinase based antigen and antibody capture enzyme immuno-assays. Mycobact Dis.

[22] Wayengera M. Diagnosis of incipient *Mycobacterium tuberculosis* (*M*. *tb*) infections using antibody based detection of *M*. *tb* thymidylate kinase: 5th IAS Conference on HIV Pathogenesis and Treatment. Abstract no. TUPEB123: Capetown; 2011.

[23] Wayengera M. TB growth and proliferation biomarkers: *Mycobacterium tuberculosis* thymidylate kinase as a candidate prototype. Keystone Symposia: overcoming the crisis of TB and AIDS (T2): Arusha; 2010.

[24] Zhang Q, Wang P, Kim Y, Haste-Andersen P, Beaver J, Bourne PE, Bui HH, Buus S, Frankild S, Greenbaum J, Lund O, Lundegaard C, Nielsen M, Ponomarenko J, Sette A, Zhu Z, Peters B (2008). Immune epitope database analysis resource (IEDB-AR). Nucleic Acids Res..

[25] Korber B, LaBute M, Yusim K (2006). Immunoinformatics comes of age. PLoS Comput Biol.

[26] Larsen JE, Lund O, Nielsen M (2006). Improved method for predicting linear B-cell epitopes. Immunome Res.

[27] Haste Andersen P, Nielsen M, Lund O (2006). Prediction of B-cell epitopes using protein 3D structures. Protein Sci.

[28] Altschul SF, Madden TL, Schäffer AA, Zhang J, Zhang Z, Miller W, Lipman DJ (1997). Gapped BLAST and PSI-BLAST: a new generation of protein database search programs. Nucleic Acids Res.

[29] Marchler-Bauer A, Zheng C, Derbyshire MK, De Weese-Scott C, Fong JH, Geer LY, Geer RC, Gonzales NR, Gwadz M, Hurwitz DI, Jackson JD, Ke Z, Lanczycki CJ, Lu F, Marchler GH, Mullokandov M, Omelchenko MV, Robertson CL, Song JS, Thanki N, Yamashita RA, Zhang D, Zhang N, Zheng C, Bryant SH (2011). CDD: a conserved domain database for the functional annotation of proteins. Nucleic Acids Res..

