# Supplementary material for: Immuno-diagnosis of Mycobacterium tuberculosis in sputum, and reduction of timelines for its positive cultures to within 3 h by pathogen-specific thymidylate kinase expression assays
Source: BMC Res Notes. 2017 Aug 8;10:368. doi: 10.1186/s13104-017-2649-y (PMC5549350; doi:10.1186/s13104-017-2649-y)
Supplement: Supplementary file 1 — Additional file 1. This file offers details of recombinant TMKmt cloning, expression and purification in E. coli BL21 (DE). [file 13104_2017_2649_MOESM1_ESM.pdf]

Certificate of Analysis

Order Number: 520141-4

Protein Name: Thymidylate kinase

Shipping Conditions: Dry Ice

Lot Number: 520141S04/P20011412

For research use only

860 Centennial Ave., Piscataway, NJ 08854, USA
Toll-Free: 1-877-436-7274 Tel: 1-732-885-9188 Fax: 1-732-210-0262 Email: order@genscript.com Web: www.genscript.com

INDEX

Certificate of Analysis..3

SDS-PAGE Analysis..4

Quantification--BSA Standard Curve...4

Packing List..5

Appendix..6

DNA Sequence...................... 6

Derived Protein Sequence...7

General procedure...8

For research use only

860 Centennial Ave., Piscataway, NJ 08854, USA

Toll-Free: 1-877-436-7274 Tel: 1-732-885-9188 Fax: 1-732-210-0262 Email: order@genscript.com Web: www.genscript.com

Certificate of Analysis

**Order Number:**	520141-4
**Protein Name:**	Thymidylate kinase
**Shipping Conditions:**	Dry Ice
**Lot Number:**	520141S04/P20011412

**Expression System:**	*E.coli*
**Purification:**	Protein was obtained from inclusion bodies

**Package:**	5.00 mg. 1.00 ml/tube, 5 tubes
**Concentration:**	1 mg/ml, as determined by Bradford protein assay with BSA as a standard
**Purity:**	About 85% as estimated by densitometric analysis of the Coomassie Blue-stained SDS-PAGE gel
**Sterility:**	Sterilized via a 0.22 μm filter and packaged aseptically
**Storage and Handling:**	Store at -80°C. Aliquots should be stored at the same temperature after first use to avoid multiple freeze-thaws
**Storage Buffer:**	50 mM Tris-HCl, 150 mM NaCl, 10% Glycerol, 500 mM L-Arginine, pH 8.0

For research use only

860 Centennial Ave., Piscataway, NJ 08854, USA
Toll-Free: 1-877-436-7274 Tel: 1-732-885-9188 Fax: 1-732-210-0262 Email: order@genscript.com Web: www.genscript.com

SDS-PAGE Analysis:

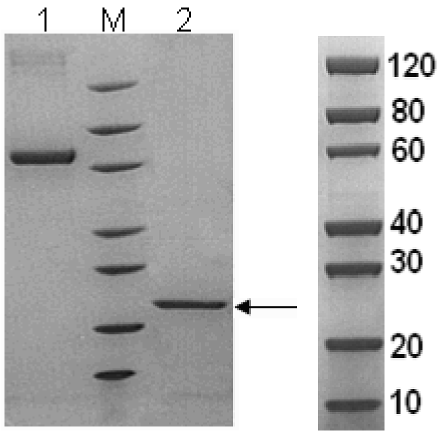

Fig.1 SDS-PAGE analysis
Lane 1: BSA (2.00 μg)
Lane 2: Thymidylate kinase (2.00 μg)

The SDS-PAGE was run on 4%~20% gradient gel, followed by Coomassie Blue staining
Protein Marker M: GenScript, Cat. No. M00516

Quantification--BSA Standard Curve:

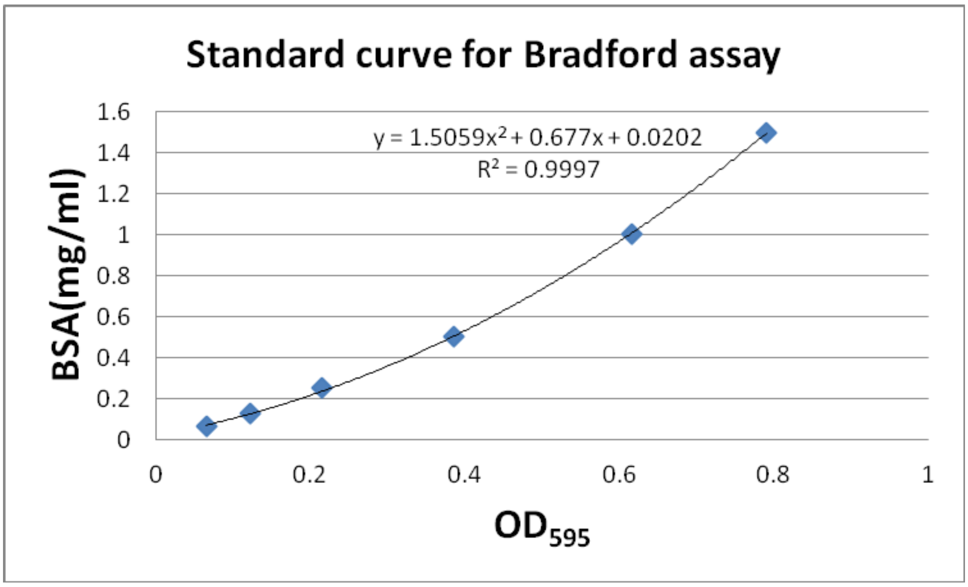

Sample	Abs at 595 nm(diluted 2 times)	Protein (mg/ml)
Thymidylate kinase	0.383	1 mg/ml

Packing List of 520141-4

Protein (Store at -80°C)

Protein name: Thymidylate kinase
tag free
Purity: >85%
Concentration: 1 mg/ml
Total: 5.00 mg. 1.00 ml/tube, 5 tubes

Certified by: Bollu **Date:** ***12/29/2014***

For research use only

860 Centennial Ave., Piscataway, NJ 08854, USA
Toll-Free: 1-877-436-7274 Tel: 1-732-885-9188 Fax: 1-732-210-0262 Email: order@genscript.com Web: www.genscript.com

Appendix

DNA Sequence (Sequencing Confirmed):

```
  1  ATGCTGATTG  CTATTGAAGG  TGTTGATGGT  GCTGGTAAAC  GTACGCTGGT  TGAAAAACTG
 61  TCTGGTGCTT  TCCGTGCGGC  GGGTCGTAGC  GTGGCTACCC  TGGCGTTTCC  GCGCTATGGC
121  CAGAGTGTTG  CGGCCGATAT  TGCAGCTGAA  GCCCTGCATG  GCGAACACGG  TGACCTGGCA
181  AGCTCTGTCT  ATGCTATGGC  AACCCTGTTC  GCACTGGATC  GTGCAGGTGC  AGTGCATACG
241  ATTCAGGGCC  TGTGCCGCGG  TTACGATGTG  GTTATCCTGG  ACCGTTATGT  TGCCAGTAAC
301  GCGGCCTACT  CCGCAGCTCG  TCTGCACGAA  AATGCAGCCG  GTAAAGCAGC  TGCATGGGTC
361  CAGCGCATCG  AATTTGCGCG  TCTGGGTCTG  CCGAAACCGG  ATTGGCAAGT  TCTGCTGGCC
421  GTCTCAGCAG  AACTGGCTGG  TGAACGTTCG  CGTGGTCGTG  CACAGCGTGA  CCCGGGTCGT
481  GCTCGTGATA  ACTATGAACG  CGACGCGGAA  CTGCAGCAAC  GTACGGGTGC  AGTGTACGCA
541  GAACTGGCAG  CACAAGGTTG  GGGCGGTCGT  TGGCTGGTCG  TGGGTGCTGA  TGTGGACCCG
601  GGCCGTCTGG  CTGCAACCCT  GGCTCCGCCG  GATGTGCCGT  CG
```


For research use only
860 Centennial Ave., Piscataway, NJ 08854, USA
Toll-Free: 1-877-436-7274 Tel: 1-732-885-9188 Fax: 1-732-210-0262 Email: order@genscript.com Web: www.genscript.com

Derived Protein Sequence:

Theoretical Isoelectric Point	Theoretical Molecular Weight
7.66	22635.0 Da

```
  1  MLIAIEGVDG  AGKRTLVEKL  SGAFRAAGRS  VATLAFPRYG  QSVAADIAAE  ALHGEHGDLA
 61  SSVYAMATLF  ALDRAGAVHT  IQGLCRGYDV  VILDRYVASN  AAYSAARLHE  NAAGKAAAWV
121  QRIEFARLGL  PKPDWQVLLA  VSAELAGERS  RGRAQRDPGR  ARDNYERDAE  LQQRTGAVYA
181  ELAAQGWGGR  WLVVGADVDP  GRLAATLAPP  DVPS
```


860 Centennial Ave., Piscataway, NJ 08854, USA
Toll-Free: 1-877-436-7274 Tel: 1-732-885-9188 Fax: 1-732-210-0262 Email: order@genscript.com Web: www.genscript.com

1. Gene Synthesis and Subcloning:

After customer's approval, target DNA sequence 520141-4(Thymidylate kinase) was designed, synthesized with related tags for each construct to facilitate the purification. The complete sequence was subcloned into target vectors for *E. coli* expression.

2. Expression Evaluation:

E. coli BL21 (DE3) was transformed with recombinant plasmids. A single colony was inoculated into medium containing ampicillin or kanamycin; cultures were incubated in 37 °C at 200 rpm. IPTG was introduced for induction. SDS-PAGE was used to monitor the expression.

3. Purification and Analysis:

Cells were harvested by centrifugation. Cell pellets were lysed by sonication, and then precipitate after centrifugation was dissolved using urea. Fractions were pooled and refolded followed by 0.22 µm filter sterilization. Proteins were analyzed by MALDI-TOF and SDS-PAGE by using standard protocols for molecular weight and purity measurements. The concentration was determined by Bradford protein assay with BSA as a standard.

860 Centennial Ave., Piscataway, NJ 08854, USA
Toll-Free: 1-877-436-7274 Tel: 1-732-885-9188 Fax: 1-732-210-0262 Email: order@genscript.com Web: www.genscript.com

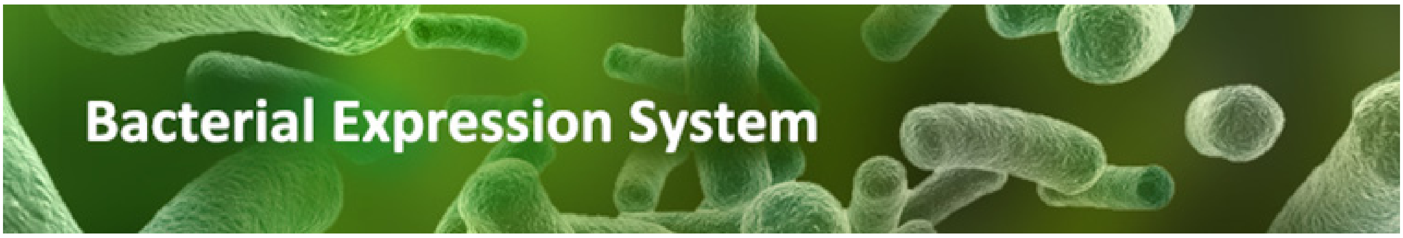

BacPower™ Guaranteed Package

- Gene synthesis is included in the package – no additional cost to you
- Guarantees the amount of custom protein – no cost to you if project fails*
- Guarantees the purity of custom protein – no cost to you if desired purity is not achieved*
- Fast turnaround time - as little as 6 weeks

For more details, please visit http://www.genscript.com/guaranteed_package_promotion.html

BacPower™ Customized Protein Service

- Flexible and affordable service tailored to meet your demands
- Fast turnaround - as little as 4 weeks to get your purified proteins
- State-of-the-art technologies - OptimumGene™ gene design& codon optimization software, BacPower™ bacterial expression system, and FoldArt™ protein refolding technology
- One-stop service platform from gene synthesis, cloning, protein expression to antibody production, structure biology and assay development

For more details, please visit http://www.genscript.com/bacterial_protein_customized_service.html

Fast gene-to-protein™

- Guarantees 3 mg of custom protein
- Guarantees 75% or 85% purity of custom protein
- Fastest turnaround - as little as 4 weeks from sequence to purified protein

For more details, please visit http://www.genscript.com/fast_gene_to_protein.html

We also provide high capacity fermentation [2000L] service for your large scale protein production needs.

*Certain rules may apply, please check our website for details

860 Centennial Ave., Piscataway, NJ 08854, USA
Toll-Free: 1-877-436-7274 Tel: 1-732-885-9188 Fax: 1-732-210-0262 Email: order@genscript.com Web: www.genscript.com
