# Supplementary figures and images for: Immuno-diagnosis of Mycobacterium tuberculosis in sputum, and reduction of timelines for its positive cultures to within 3 h by pathogen-specific thymidylate kinase expression assays
Source: BMC Res Notes. 2017 Aug 8;10:368. doi: 10.1186/s13104-017-2649-y (PMC5549350; doi:10.1186/s13104-017-2649-y)

## Slide 1
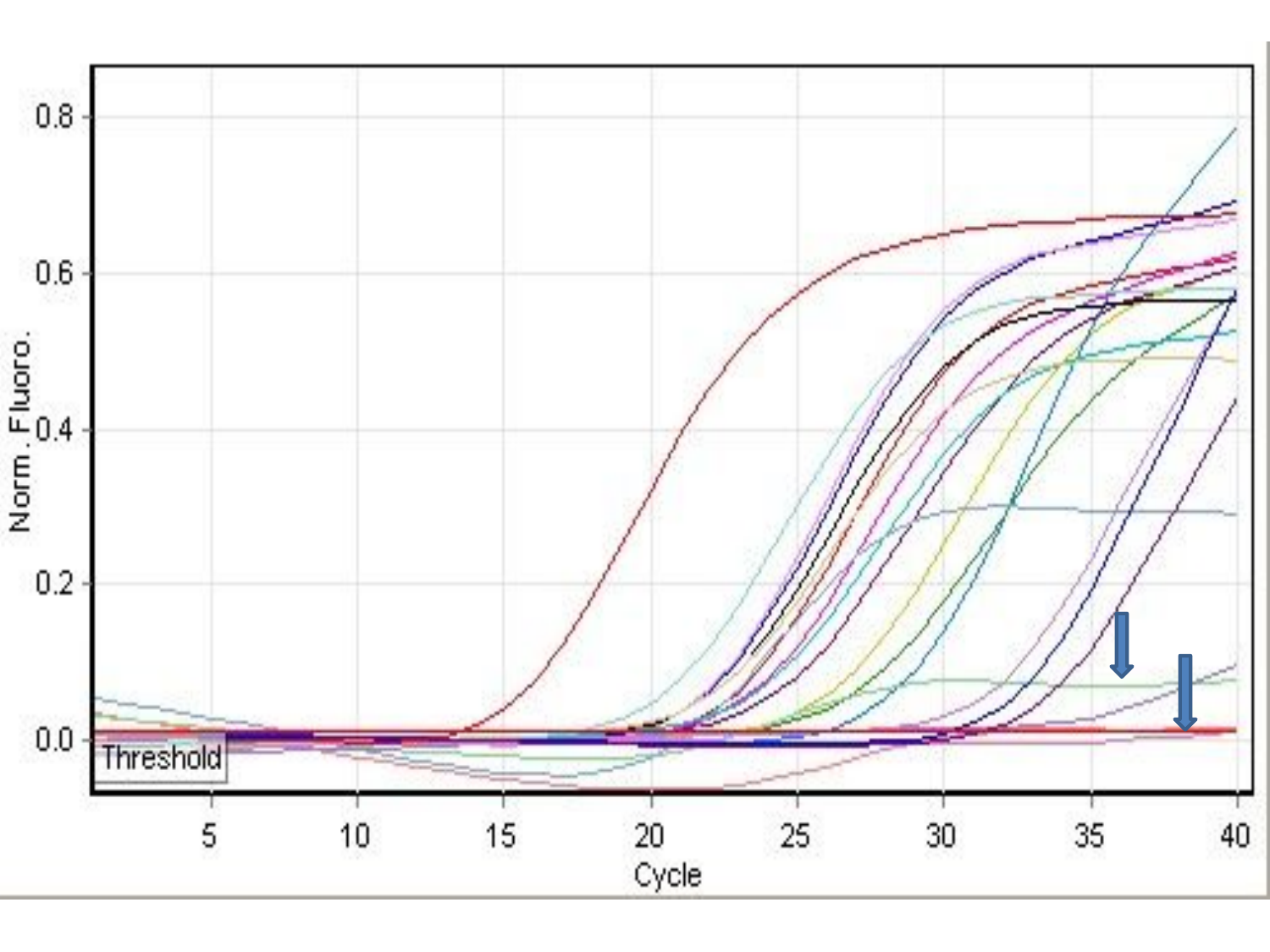

Supplement: Supplementary file 3 — Additional file 3. This figures shows optimization curves of qRT-PCR for TMKmt and M.tb Pol1A. [file 13104_2017_2649_MOESM3_ESM.pptx]

## Slide 1
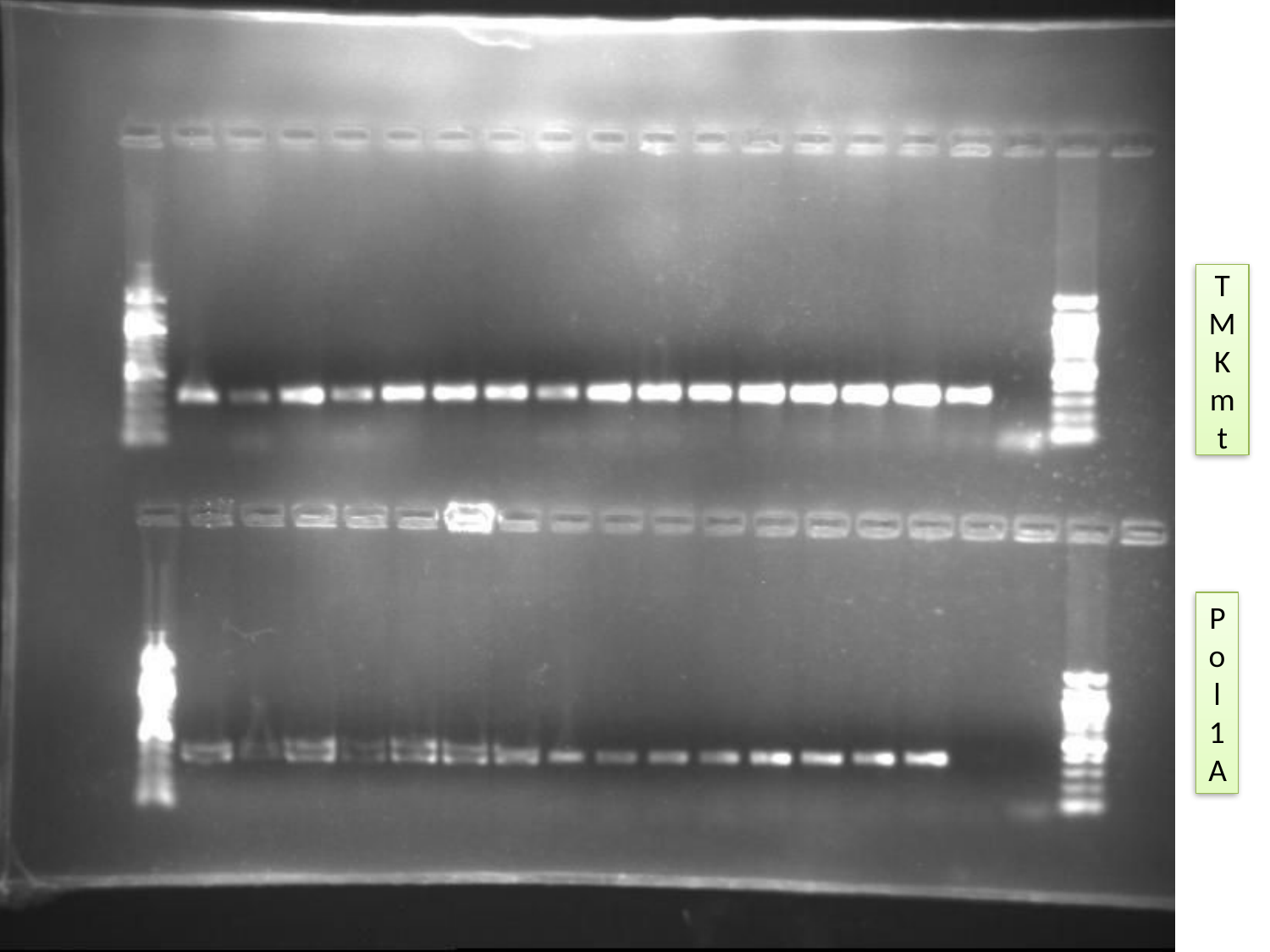

TMKmt
Pol1A

Supplement: Supplementary file 4 — Additional file 4. This figures depicts gel electrophoris analysis of PCR amplicons of TMKmt and M.tb Pol1A. Note the 22kDA placement of the TMKmt amplicon relative to protein marker M. [file 13104_2017_2649_MOESM4_ESM.pptx]

## Slide 1
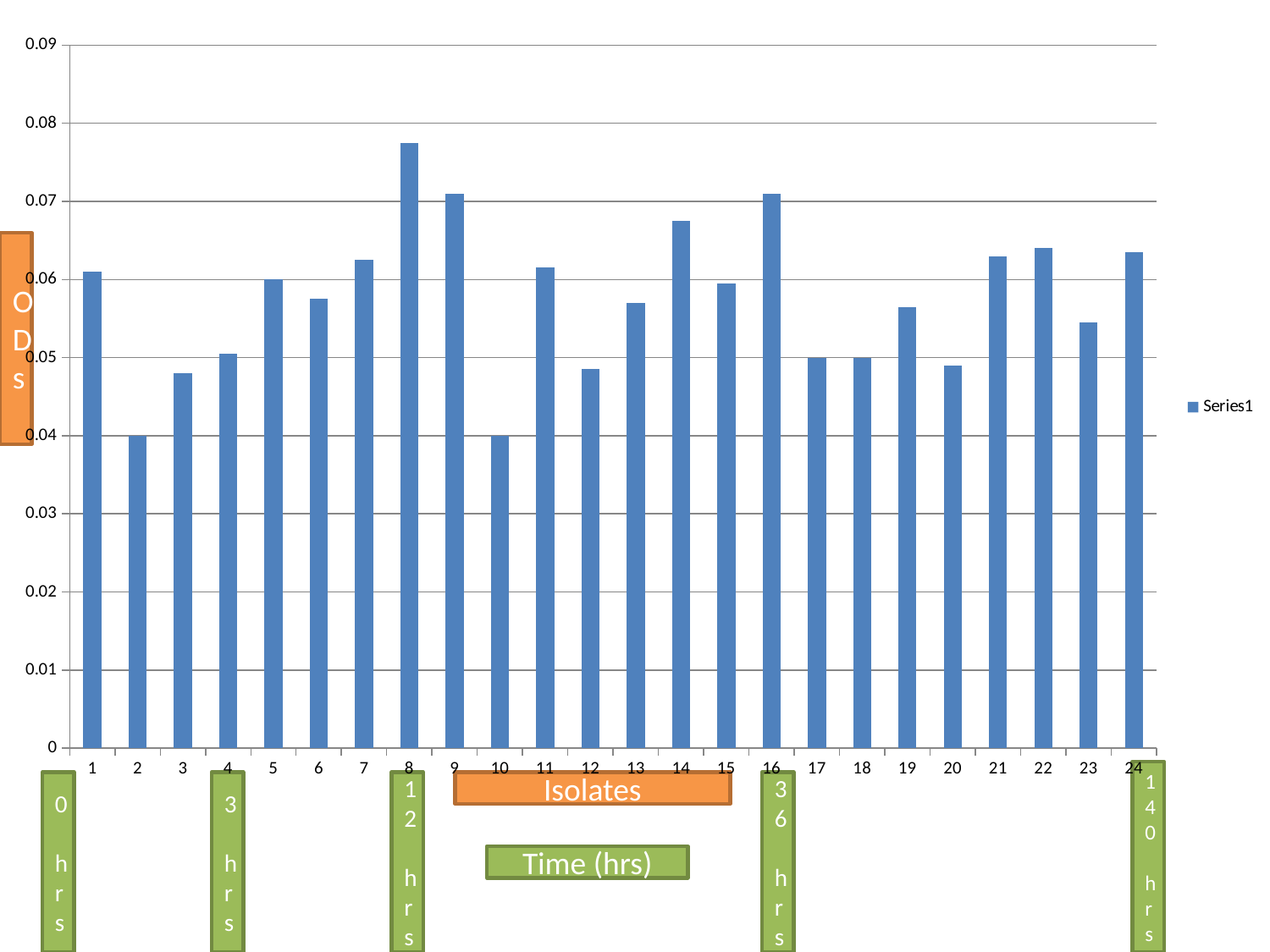

### Chart
| Category | |
|---|---|ODs
140
hrs
0 hrs
3
hrs
12
hrs
Isolates
36
hrs
Time (hrs)

Supplement: Supplementary file 5 — Additional file 5. This figure shows TMKmt Ag levels in serial dilutions of 1+ acid fast baccili ladden patient sputum sample detected by PAb-0656. Direct Enzyme Immuno-assay (EIA) were conducted using two custom polyclonal antibodies (PAb-0655 and PAb-0656) in the 1x10^-1^, 1x10^-2^, 1x10^-3^, 1x10^-4^, 1x10^-4^, 1x10^-5^, 1x10^-6^ dilutions of sputum containing (considering the initial 1+ AFB sputum with 105 CFUs/ml) approximately 104, 103, 102, 10, 1, and 0.1 CFUs/ml of acid fast bacilli are respectively. Note that relative to the blank, TMKmt Ag was detected upto 1x10^-4^ to 1x10^-5^ (containing 10 and 1 CFUs/ml) dilutions of a microscopically designated 1+ (est. Acid Fast Bacillary load of 1x10^5^) patient sample by PAb-0655 and PAb-0656, respectively. [file 13104_2017_2649_MOESM5_ESM.pptx]

## Slide 1
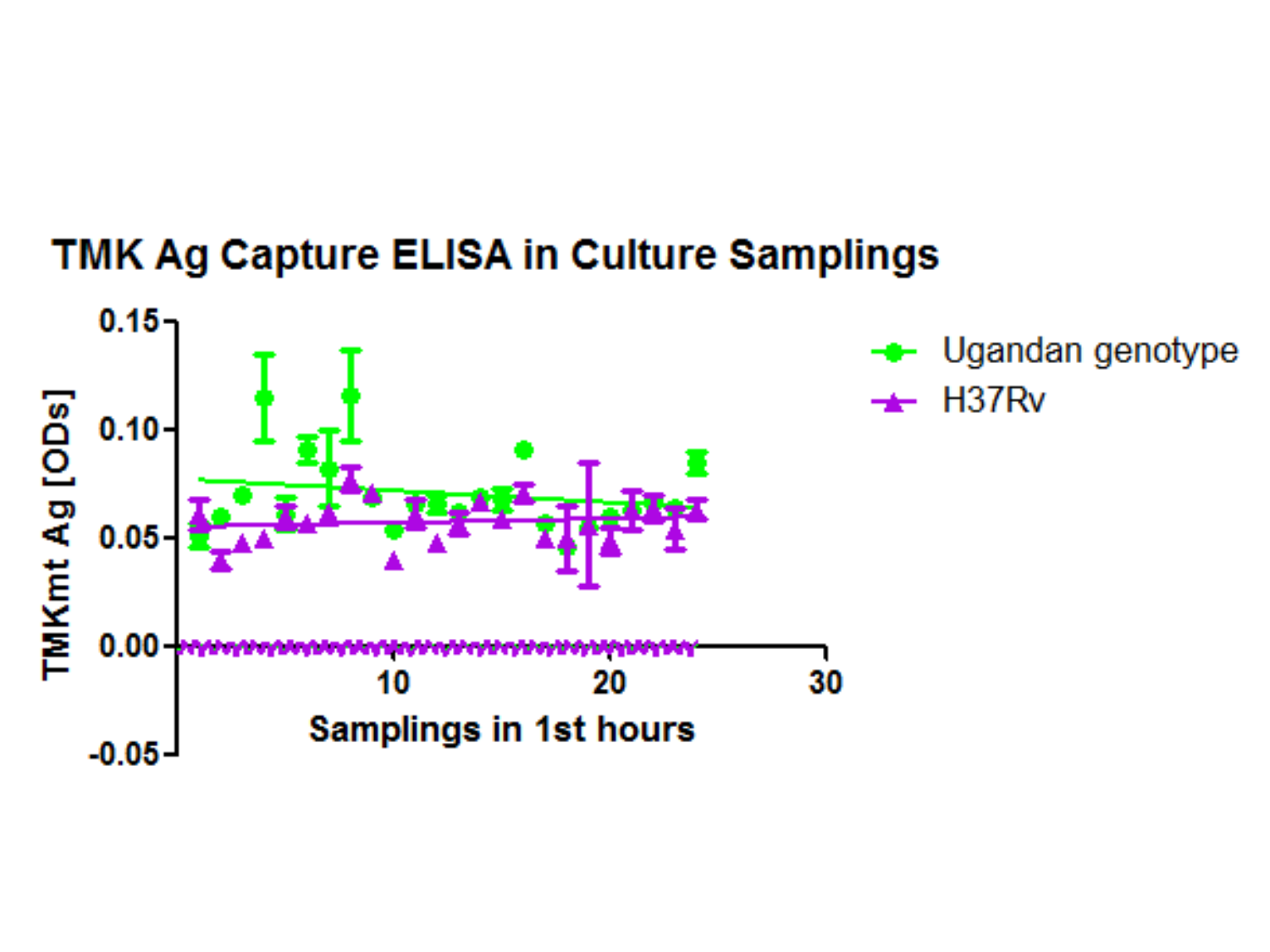

Supplement: Supplementary file 6 — Additional file 6. This figure shows the GraphPad combined TMKmt antigen expression profile among pure cultures of Uganda genotype 1 detected by PAb-0655 and PAb-0656. In comparision, the cyclic pattern for expression of TMKmt Ag among H37RV (not shown), was different from the Ugandan genotype 1. Specifically, H37RV demonstrated an early but short lived spike in TMKmt Ag levels between 0 and 3 hours post innoculation, with the next spike emerging at 12 hours. This early spike in H37RV TMKmt Ag expression profiles, may explain why this lab strain grows faster than pathogenic strains. Important to note is that, regardless of these differences in cyclic patterns of TMKmt Ag expression, the Ugandan genotype displayed higher amplitudes of TMKmt mRNA expression. [file 13104_2017_2649_MOESM6_ESM.pptx]

## Slide 1
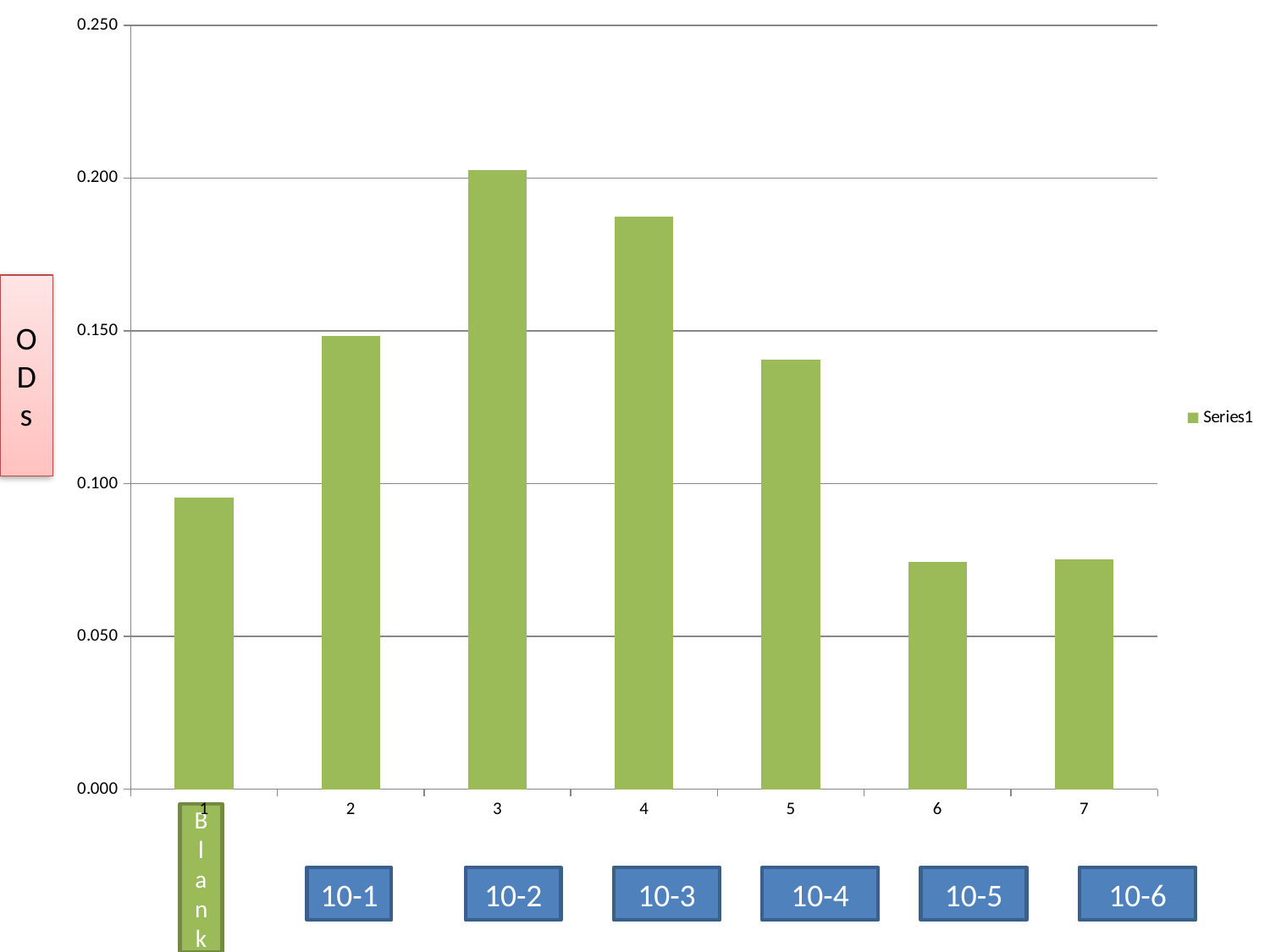

### Chart
| Category | |
|---|---|ODs
Blank
10-1
10-2
10-3
10-4
10-5
10-6

Supplement: Supplementary file 8 — Additional file 8. This figure shows TMKmt Ag levels in serial dilutions of 1+ acid fast baccili ladden patient sputum sample detected by PAb-0656 conjugate. Direct Enzyme Immuno-assay (EIA) were conducted using PAb-0656 conjuagte in the 1x10^-1^, 1x10^-2^, 1x10^-3^, 1x10^-4^, 1x10^-5^, 1x10^-6^ dilutions of sputum containing (considering the initial 1+ AFB sputum with 105 CFUs/ml) approximately 104, 103, 102, 10, 1 and 0.1 CFUs/ml of acid fast bacilli are respectively. Note that relative to the blank, TMKmt Ag was detected upto 1x10^-5^ (containing 1 CFUs/ml) dilutions of a microscopically designated 1+ (est. Acid Fast Bacillary load of 1x10^5^) patient sample by PAb-0656. [file 13104_2017_2649_MOESM8_ESM.pptx]

## Slide 1
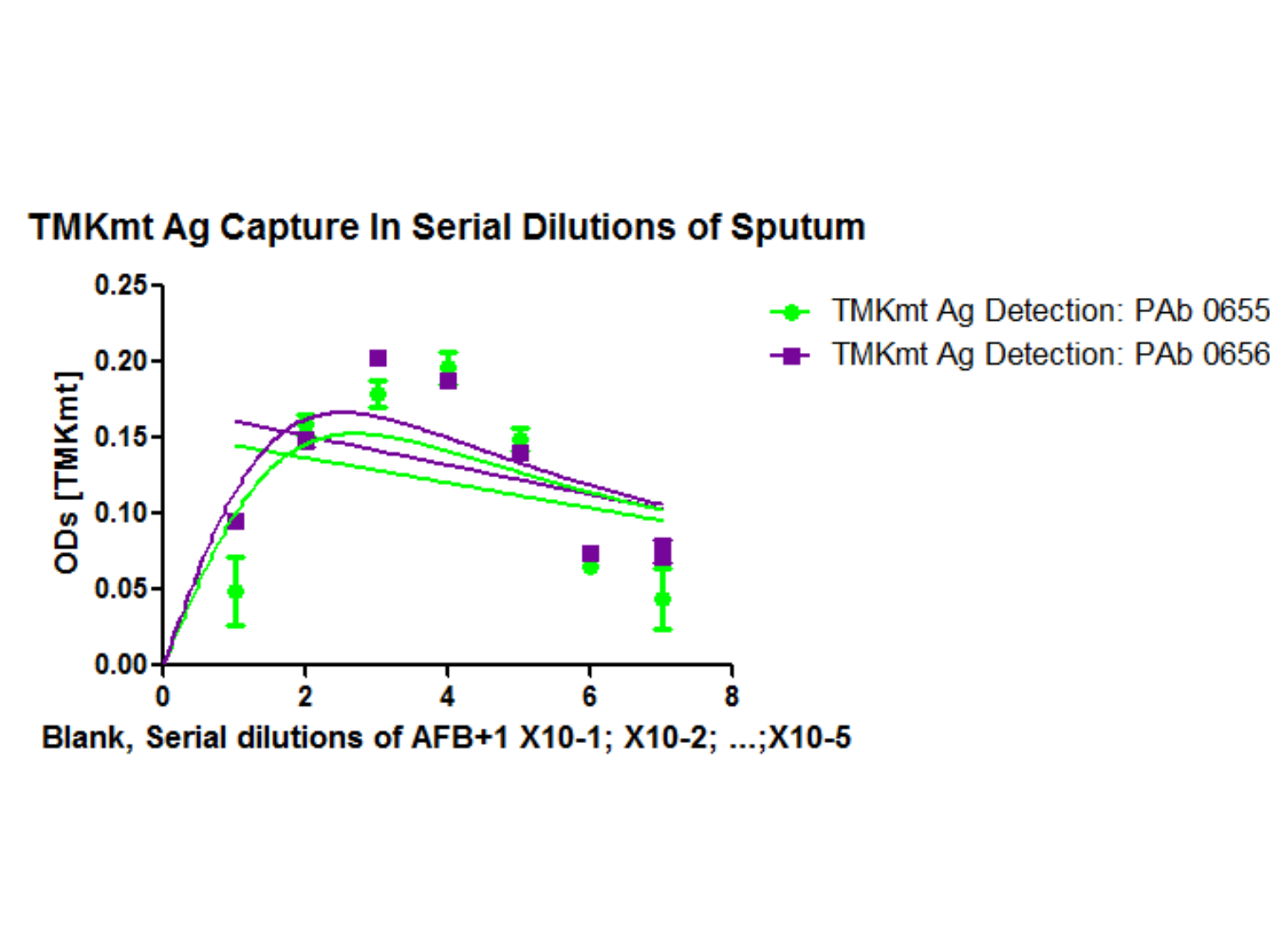

Supplement: Supplementary file 9 — Additional file 9. This figure shows GraphPad combined TMKmt Ag levels in serial dilutions of 1+ acid fast baccili ladden patient sputum sample detected by PAb-0655 and PAb-0656 conjugates, respectively. Direct Enzyme Immuno-assay (EIA) were conducted using either PAb-0655 or PAb-0656 conjuagte in the 1x10^-1^, 1x10^-2^, 1x10^-3^, 1x10^-4^, 1x10^-5^, 1x10^-6^ dilutions of sputum containing (considering the initial 1+ AFB sputum with 105 CFUs/ml) approximately 104, 103, 102, 10, 1, and 0.1 CFUs/ml of acid fast bacilli are respectively. Note that relative to the blank, TMKmt Ag was detected upto 1x10^-4^ and 1x10^-5^ (containing 10 and 1 CFUs/ml) dilutions of a microscopically designated 1+ (est. Acid Fast Bacillary load of 1x10^5^) patient sample by PAb-0655 and PAb-0656, respectively. [file 13104_2017_2649_MOESM9_ESM.pptx]
