# Supplementary material for: Immuno-diagnosis of Mycobacterium tuberculosis in sputum, and reduction of timelines for its positive cultures to within 3 h by pathogen-specific thymidylate kinase expression assays
Source: BMC Res Notes. 2017 Aug 8;10:368. doi: 10.1186/s13104-017-2649-y (PMC5549350; doi:10.1186/s13104-017-2649-y)
Supplement: Supplementary file 11 — Additional file 11. This file offers details of cross validated by BLAST across the NCBI microbial- databases. [file 13104_2017_2649_MOESM11_ESM.pdf]

BLAST ®

Basic Local Alignment Search Tool

NCBI/ BLAST/ Microbes/ **Formatting Results - 0V0XK1R5015**
Formatting options
Download
Blast report description

Protein Sequence (27 letters)

RID 0V0XK1R5015 (Expires on 09-09 18:23 pm)

Query ID lcl|71609
Description None
Molecule type amino acid
Query Length 27

Database Name Microbial proteins
Description
Program BLASTP 2.2.29+

New DELTA-BLAST, a more sensitiv

Graphic Summary

Putative conserved domains have been detected, click on the image below for detailed results.

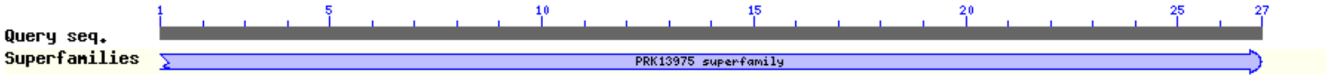

Distribution of 100 Blast Hits on the Query Sequence

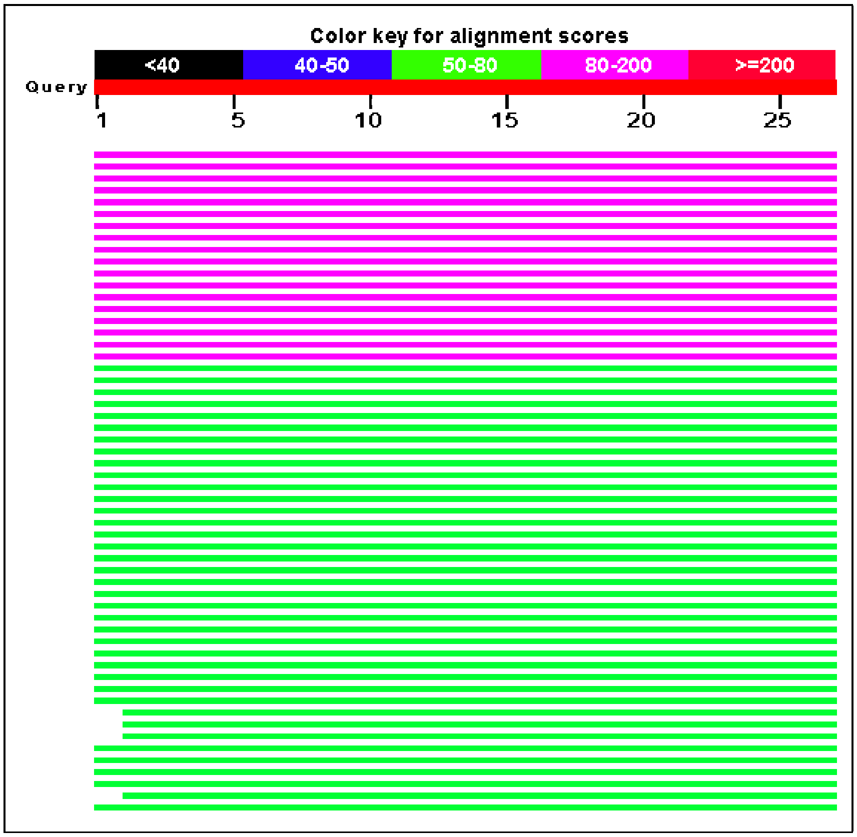

Descriptions

Sequences producing significant alignments:

Description	Max score	Total score	Query cover	E value	Ident	Accession
thymidylate kinase Tmk [Mycobacterium tuberculosis UT0124]	90.1	90.1	100%	5e-20	100%	KBM28427.1
thymidylate kinase [Mycobacterium tuberculosis]	90.1	90.1	100%	3e-19	100%	AIH62975.1
thymidylate kinase [Mycobacterium tuberculosis]	90.1	90.1	100%	3e-19	100%	AIH98343.1
thymidylate kinase [Mycobacterium bovis]	90.1	90.1	100%	3e-19	100%	WP_024459054.1
thymidylate kinase [Mycobacterium tuberculosis]	90.1	90.1	100%	4e-19	100%	AIH23782.1
thymidylate kinase [Mycobacterium tuberculosis]	90.1	90.1	100%	4e-19	100%	WP_003904223.1
thymidylate kinase Tmk [Mycobacterium tuberculosis BTB11-133]	90.1	90.1	100%	4e-19	100%	KCQ37814.1
thymidylate kinase Tmk [Mycobacterium tuberculosis BTB07-206]	90.1	90.1	100%	4e-19	100%	KCN91622.1
thymidylate kinase Tmk [Mycobacterium tuberculosis M1520]	90.1	90.1	100%	4e-19	100%	KBA42335.1
thymidylate kinase Tmk [Mycobacterium tuberculosis M1461]	90.1	90.1	100%	4e-19	100%	KBA29358.1
thymidylate kinase Tmk [Mycobacterium tuberculosis M1417]	90.1	90.1	100%	4e-19	100%	KAZ51277.1
MULTISPECIES: thymidylate kinase [Mycobacterium tuberculosis complex]	90.1	90.1	100%	4e-19	100%	WP_003417036.1
Putative thymidylate kinase TmK (dTMP kinase) (thymidylic acid kinase) (TMPK) [Mycobacterium canettii]	90.1	90.1	100%	4e-19	100%	WP_015294285.1
Putative thymidylate kinase TmK (dTMP kinase) (thymidylic acid kinase) (TMPK) [Mycobacterium canettii]	90.1	90.1	100%	4e-19	100%	WP_015291311.1
Putative thymidylate kinase TmK (dTMP kinase) (thymidylic acid kinase) (TMPK) [Mycobacterium canettii]	90.1	90.1	100%	4e-19	100%	WP_015303784.1
thymidylate kinase [Mycobacterium tuberculosis]	90.1	90.1	100%	4e-19	100%	WP_015456493.1
thymidylate kinase [Mycobacterium canettii]	90.1	90.1	100%	4e-19	100%	WP_014001640.1
thymidylate kinase [Mycobacterium tuberculosis]	87.6	87.6	100%	3e-18	96%	WP_024748275.1
hypothetical protein [Mycobacterium avium]	64.3	64.3	100%	7e-11	74%	WP_023877133.1
Thymidylate kinase [Mycobacterium marinum]	65.5	65.5	100%	1e-10	78%	WP_020727270.1
Thymidylate kinase [Mycobacterium sp. 012931]	65.5	65.5	100%	1e-10	78%	WP_020788368.1
thymidylate kinase Tmk [Mycobacterium liflandii]	65.5	65.5	100%	1e-10	78%	WP_015354855.1
thymidylate kinase [Mycobacterium marinum]	65.5	65.5	100%	1e-10	78%	WP_012393166.1
thymidylate kinase [Mycobacterium ulcerans]	65.5	65.5	100%	1e-10	78%	WP_011740536.1
thymidylate kinase [Mycobacterium kansasii 732]	64.7	64.7	100%	2e-10	74%	EUA14516.1
thymidylate kinase [Mycobacterium sp. TKK-01-0059]	64.3	64.3	100%	3e-10	74%	KEF99753.1
thymidylate kinase [Mycobacterium avium subsp. hominissuis 3388]	64.3	64.3	100%	3e-10	74%	KDO94907.1
thymidylate kinase [Mycobacterium avium]	64.3	64.3	100%	3e-10	74%	WP_003874565.1
thymidylate kinase [Mycobacterium avium]	64.3	64.3	100%	3e-10	74%	WP_023864921.1
thymidylate kinase [Mycobacterium avium]	64.3	64.3	100%	3e-10	74%	WP_023883946.1
thymidylate kinase [Mycobacterium avium subsp. hominissuis TH135]	64.3	64.3	100%	3e-10	74%	BAN32696.1
thymidylate kinase [Mycobacterium indicus pranii]	64.3	64.3	100%	3e-10	74%	WP_014942809.1
thymidylate kinase [Mycobacterium intracellulare]	64.3	64.3	100%	3e-10	74%	WP_009957361.1
thymidylate kinase [Mycobacterium avium]	64.3	64.3	100%	3e-10	74%	WP_009978739.1
thimidylate kinase [Mycobacterium avium subsp. paratuberculosis]	64.3	64.3	100%	3e-10	74%	AAL10206.1
thymidylate kinase [Mycobacterium genavense]	61.7	61.7	100%	2e-09	74%	WP_025737680.1

Description	Max score	Total score	Query cover	E value	Ident	Accession
thymidylate kinase [Segniliparus rotundus]	61.3	61.3	100%	3e-09	74%	WP_013139117.1
thymidylate kinase [Mycobacterium gastri 'Wayne']	61.3	61.3	100%	3e-09	70%	ETW22207.1
thymidylate kinase [Mycobacterium parascrofulaceum]	60.9	60.9	100%	4e-09	70%	WP_007168096.1
thymidylate kinase [Mycobacterium kansasii]	60.4	60.4	100%	5e-09	70%	WP_023371563.1
thymidylate kinase [Mycobacterium intracellulare MIN_061107_1834]	59.2	59.2	100%	1e-08	70%	ETZ31677.1
thymidylate kinase [Mycobacterium intracellulare MIN_052511_1280]	59.2	59.2	100%	1e-08	70%	ETZ27386.1
thymidylate kinase [Mycobacterium avium MAV_120809_2495]	59.2	59.2	100%	1e-08	70%	ETZ47660.1
thymidylate kinase [Mycobacterium sp. H4Y]	59.2	59.2	100%	1e-08	70%	WP_008259770.1
thymidylate kinase [Mycobacterium sp. MOTT36Y]	59.2	59.2	100%	1e-08	70%	WP_014712266.1
thymidylate kinase [Mycobacterium intracellulare]	59.2	59.2	100%	1e-08	70%	WP_014385672.1
thymidylate kinase [Mycobacterium intracellulare]	59.2	59.2	100%	1e-08	70%	WP_014380959.1
thymidylate kinase [Mycobacterium tuberculosis TKK-01-0051]	59.2	59.2	96%	1e-08	73%	KBZ68881.1
thymidylate kinase [Mycobacterium colombiense]	59.2	59.2	96%	1e-08	73%	WP_007773133.1
thymidylate kinase [Mycobacterium yongonense]	57.5	57.5	96%	5e-08	69%	WP_020823379.1
thymidylate kinase [Mycobacterium intracellulare]	55.8	55.8	100%	2e-07	67%	WP_014383184.1
thymidylate kinase [Mycobacterium triplex]	54.1	54.1	100%	7e-07	67%	CDO87354.1
thymidylate kinase [Gordonia otitidis]	52.4	52.4	100%	2e-06	67%	WP_007238888.1
thymidylate kinase [Segniliparus rugosus]	52.0	52.0	100%	3e-06	70%	WP_007470136.1
thymidylate kinase [Turicella otitidis]	51.5	51.5	96%	4e-06	65%	WP_004601034.1
thymidylate kinase [Mycobacterium abscessus]	51.5	51.5	100%	4e-06	59%	WP_005117148.1
thymidylate kinase [Mycobacterium abscessus]	51.5	51.5	100%	4e-06	59%	WP_005102107.1
thymidylate kinase [Mycobacterium abscessus]	51.5	51.5	100%	5e-06	59%	WP_025239600.1
thymidylate kinase [Mycobacterium abscessus]	51.5	51.5	100%	5e-06	59%	WP_005088442.1
thymidylate kinase [Mycobacterium abscessus]	51.5	51.5	100%	5e-06	59%	WP_005080864.1
thymidylate kinase [Mycobacterium abscessus]	51.5	51.5	100%	5e-06	59%	WP_005070201.1
thymidylate kinase [Mycobacterium abscessus]	51.5	51.5	100%	5e-06	59%	WP_005056276.1
thymidylate kinase [Mycobacterium abscessus]	51.5	51.5	100%	5e-06	59%	WP_005111910.1
thymidylate kinase [Rhodococcus sp. UNC23MFCrub1.1]	51.1	51.1	96%	6e-06	69%	WP_027506870.1
MULTISPECIES: thymidylate kinase [Mycobacterium]	50.7	50.7	100%	9e-06	59%	WP_025088603.1
thymidylate kinase [Gordonia sputi]	49.8	49.8	100%	2e-05	63%	WP_005208003.1
thymidylate kinase [Mycobacterium abscessus]	49.0	49.0	100%	3e-05	59%	WP_016892938.1
thymidylate kinase [Gordonia paraffinivorans]	48.6	48.6	100%	4e-05	63%	WP_006899672.1
thymidylate kinase [Corynebacterium halotolerans]	48.1	48.1	100%	6e-05	59%	WP_015400173.1
thymidylate kinase [Mycobacterium sp. JDM601]	47.7	47.7	100%	8e-05	59%	WP_013829916.1
hypothetical protein [Gordonia kroppenstedtii]	47.7	47.7	81%	8e-05	62%	WP_018180987.1
thymidylate kinase [Mycobacterium abscessus]	47.3	47.3	100%	1e-04	56%	WP_005077366.1
thymidylate kinase [Mycobacterium leprae]	46.9	46.9	96%	1e-04	69%	WP_010907920.1
thymidylate kinase [Gordonia effusa]	46.9	46.9	100%	1e-04	63%	WP_007315852.1
thymidylate kinase [Gordonia polyisoprenivorans]	46.9	46.9	81%	1e-04	65%	WP_026919949.1
thymidylate kinase [Gordonia polyisoprenivorans]	46.9	46.9	81%	1e-04	65%	WP_006370363.1
thymidylate kinase [Dietzia cinnamea]	46.9	46.9	59%	1e-04	88%	WP_007630481.1
thymidylate kinase [Tomitella biformata]	46.4	46.4	100%	2e-04	64%	WP_024793529.1
thymidylate kinase [Gordonia terrae]	46.4	46.4	81%	2e-04	68%	WP_004023514.1
thymidylate kinase [Mycobacterium xenopi 3993]	46.0	46.0	100%	2e-04	59%	EUA32800.1
hypothetical protein [Corynebacterium capitovis]	46.0	46.0	96%	3e-04	57%	WP_018017662.1
thymidylate kinase [Mycobacterium avium]	46.0	46.0	100%	3e-04	59%	WP_019737853.1

Description	Max score	Total score	Query cover	E value	Ident	Accession
thymidylate kinase [Mycobacterium xenopi 4042]	46.0	46.0	100%	3e-04	59%	EUA06831.1
thymidylate kinase [Mycobacterium xenopi]	46.0	46.0	100%	3e-04	59%	WP_003920064.1
thymidylate kinase [Corynebacterium genitalium]	45.6	45.6	88%	4e-04	67%	WP_005286098.1
hypothetical protein [Corynebacterium doosanense]	45.2	45.2	100%	5e-04	63%	WP_018021130.1
thymidylate kinase [Corynebacterium tuberculostearicum]	45.2	45.2	100%	5e-04	59%	WP_005326584.1
thymidylate kinase [Corynebacterium pseudogenitalium]	45.2	45.2	100%	5e-04	59%	WP_005322408.1
thymidylate kinase [Rhodococcus pyridinivorans]	45.2	45.2	100%	5e-04	59%	WP_006550215.1
hypothetical protein [Corynebacterium lubricantis]	44.8	44.8	92%	7e-04	62%	WP_018296139.1
MULTISPECIES: thymidylate kinase [Corynebacterium]	44.8	44.8	100%	7e-04	59%	WP_023020774.1
thymidylate kinase [Mycobacterium neoaurum]	44.8	44.8	100%	7e-04	59%	WP_030134849.1
thymidylate kinase [Gordonia bronchialis]	44.8	44.8	92%	7e-04	60%	WP_012835337.1
hypothetical protein [Smaragdicoccus niigatensis]	44.8	44.8	96%	7e-04	58%	WP_018163288.1
thymidylate kinase [Gordonia soli]	44.3	44.3	85%	0.001	59%	WP_007617234.1
thymidylate kinase [Corynebacterium urealyticum]	44.3	44.3	100%	0.001	56%	WP_015381340.1
thymidylate kinase [Corynebacterium urealyticum]	44.3	44.3	100%	0.001	56%	WP_012359732.1
thymidylate kinase [Mycobacterium aromaticivorans JS19b1]	43.9	43.9	100%	0.001	59%	KDE98580.1
hypothetical protein [Corynebacterium pyruviciproducens]	43.5	43.5	100%	0.002	59%	WP_016458814.1
thymidylate kinase [Dietzia sp. UCD-THP]	43.5	43.5	92%	0.002	67%	WP_017836632.1

Alignments

thymidylate kinase Tmk, partial [Mycobacterium tuberculosis UT0124]
Sequence ID: **gb|KBM28427.1|** Length: 90 Number of Matches: 1
Range 1: 24 to 50

Score	Expect	Method	Identities	Positives	Gaps	Frame
90.1 bits(205)	5e-20()		27/27(100%)	27/27(100%)	0/27(0%)	

Features:

```
Query  1   ERSRGRAQRDPGRARDNYERDAELQQR  27
           ERSRGRAQRDPGRARDNYERDAELQQR
Sbjct  24  ERSRGRAQRDPGRARDNYERDAELQQR  50
```

thymidylate kinase, partial [Mycobacterium tuberculosis]
Sequence ID: **gb|AIH62975.1|** Length: 171 Number of Matches: 1
Range 1: 105 to 131

Score	Expect	Method	Identities	Positives	Gaps	Frame
90.1 bits(205)	3e-19()		27/27(100%)	27/27(100%)	0/27(0%)	

Features:

```
Query  1    ERSRGRAQRDPGRARDNYERDAELQQR  27
            ERSRGRAQRDPGRARDNYERDAELQQR
Sbjct  105  ERSRGRAQRDPGRARDNYERDAELQQR  131
```

thymidylate kinase, partial [Mycobacterium tuberculosis]
Sequence ID: **gb|AIH98343.1|** Length: 175 Number of Matches: 1
Range 1: 109 to 135

Score	Expect	Method	Identities	Positives	Gaps	Frame
90.1 bits(205)	3e-19()		27/27(100%)	27/27(100%)	0/27(0%)	

Features:

```
Query  1    ERSRGRAQRDPGRARDNYERDAELQQR  27
```

```
             ERSRGRAQRDPGRARDNYERDAELQQR
Sbjct  109   ERSRGRAQRDPGRARDNYERDAELQQR  135
```

thymidylate kinase, partial [Mycobacterium bovis]

Sequence ID: **ref**|**WP_024459054.1**| Length: 210 Number of Matches: 1
Range 1: 144 to 170

Score	Expect	Method	Identities	Positives	Gaps	Frame
90.1 bits(205)	3e-19()		27/27(100%)	27/27(100%)	0/27(0%)	

Features:

```
Query  1     ERSRGRAQRDPGRARDNYERDAELQQR  27
             ERSRGRAQRDPGRARDNYERDAELQQR
Sbjct  144   ERSRGRAQRDPGRARDNYERDAELQQR  170
```

thymidylate kinase [Mycobacterium tuberculosis]

Sequence ID: **gb**|**AIH23782.1**| Length: 214 Number of Matches: 1
Range 1: 148 to 174

Score	Expect	Method	Identities	Positives	Gaps	Frame
90.1 bits(205)	4e-19()		27/27(100%)	27/27(100%)	0/27(0%)	

Features:

```
Query  1     ERSRGRAQRDPGRARDNYERDAELQQR  27
             ERSRGRAQRDPGRARDNYERDAELQQR
Sbjct  148   ERSRGRAQRDPGRARDNYERDAELQQR  174
```
